# Supplementary material for: Addressing delayed case reporting in infectious disease forecast modeling
Source: PLoS Comput Biol. 2022 Jun 3;18(6):e1010115. doi: 10.1371/journal.pcbi.1010115 (PMC9200328; doi:10.1371/journal.pcbi.1010115)
Supplement: S1 Text — Fig A: Comparing initially reported and validation case counts for Puerto Rico dengue fever and US national influenza-like illness. In all plots, the highest (black) curve corresponds to validation case counts. The lowest (red) curve corresponds to the cases initially reported (lag = 0). Fig B: Reported ILI cases in Vermont for each calendar week in the 2017–2018 and 2018–2019 flu seasons. Data for Vermont ILI cases were downloaded on June 13th, 2021. Lines correspond to the first 35 calendar weeks in the corresponding flu season. Fig C: Estimated proportion of validation cases initially reported by state using data from the 2018–2019 season and data from the previous two seasons. Data were downloaded on June 13th, 2021. Results from the current season may be viewed as the “truth” here, but data analysis and forecasting is conducted using estimates from the previous two seasons (except for local lag-based estimation). Table A: Summary of key notation. Fig D: Diagram of Multiple Imputation Algorithm. Fig E: Percent error between truncated normal expectation and E(Nts(∞)|Nts(d)). Fig F: Comparison of regression model estimates of πts(d) from Eq 7 based on historical real-time case reporting and the observed values of πts(d) = Nts(d)/Nts(∞) for national US ILI and for dengue fever in Puerto Rico. Model-based estimates of πts(d) are obtained by fitting the regression model in Eq. h in S1 Text. to historical reporting data, excluding the most recent 16 weeks (ILI) or the most recent 6 weeks (dengue fever) from the estimation. Values along the black line indicate model-based estimates of πts(d) that closely align with observed values of πts(d). Fig G: Weighted interval scores of forecasts in the Puerto Rico dengue fever and national US influenza-like illness data across all weeks. Results for dengue fever are aggregated across each of 50 weeks in 18 seasons (1992–2009). Results for US national influenza are aggregated across 35 weeks in 8 seasons (2012–2018). The ensemble [file pcbi.1010115.s001.pdf]

Supporting Text 1 for "Addressing delayed case reporting in infectious disease forecast modeling" by

Lauren J. Beesley*[1], Dave Osthus[1], and Sara Del Valle[1]
[1]Los Alamos National Laboratory, Los Alamos, New Mexico, USA
*lvandervort@lanl.gov

Contents

1 Visualizing backfill **2**

2 Summary of notation **6**

3 Modeling available cases with an offset **7**

4 Obtaining multiple imputations for validation case counts **9**

5 Forecasting models **12**

6 Application to dengue fever and ILI data **14**

7 A note on lag scaling **30**

8 Simulations of dengue fever data **33**

9 Simulations of national US ILI data **37**

10 Extension for modeling percent ILI among outpatient visits **42**

1 Visualizing backfill

In this section, we provide some visualization of backfill/reporting delay in the exemplar datasets for dengue fever in Puerto Rico and seasonal influenza in the United States. In **Figure A**, we compare the number of cases initially reported and reported after 1 week to the validation case counts for both dengue fever and national influenza-like illness (ILI) data. **Figure B** demonstrates the large difference in reporting errors between the 2017-2018 season and the 2018-2019 season for Vermont, indicating that use of past-season data to estimate reporting factors for the 2018-2019 season may result in suboptimal performance for Vermont. This problem is not limited to Vermont. **Figure C** shows the average estimated $\hat{\pi}(0)$ (i.e., the proportion of eventually-reported validation cases reported initially) for the "current" 2018-2019 season and for the previous two seasons for each state. While Vermont has by far the largest difference in $\hat{\pi}(0)$ between seasons, many other states also had large differences in reporting practices between seasons. Naturally, this will negatively impact the performance of methods that use past-season data to estimate reporting factors in the 2018-2019 season.

Figure A: Comparing initially reported and validation case counts for Puerto Rico dengue fever and US national influenza-like illness [1]

(a) Puerto Rico dengue fever data, 2000-2008

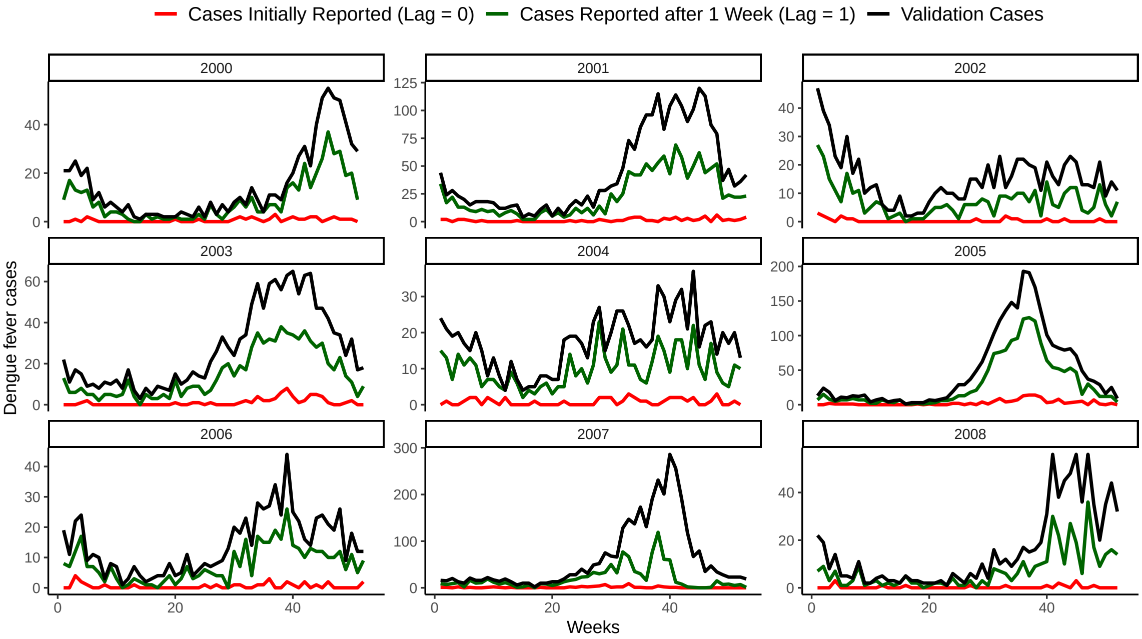

(b) National US Influenza, 2010-2011 to 2018-2019 seasons

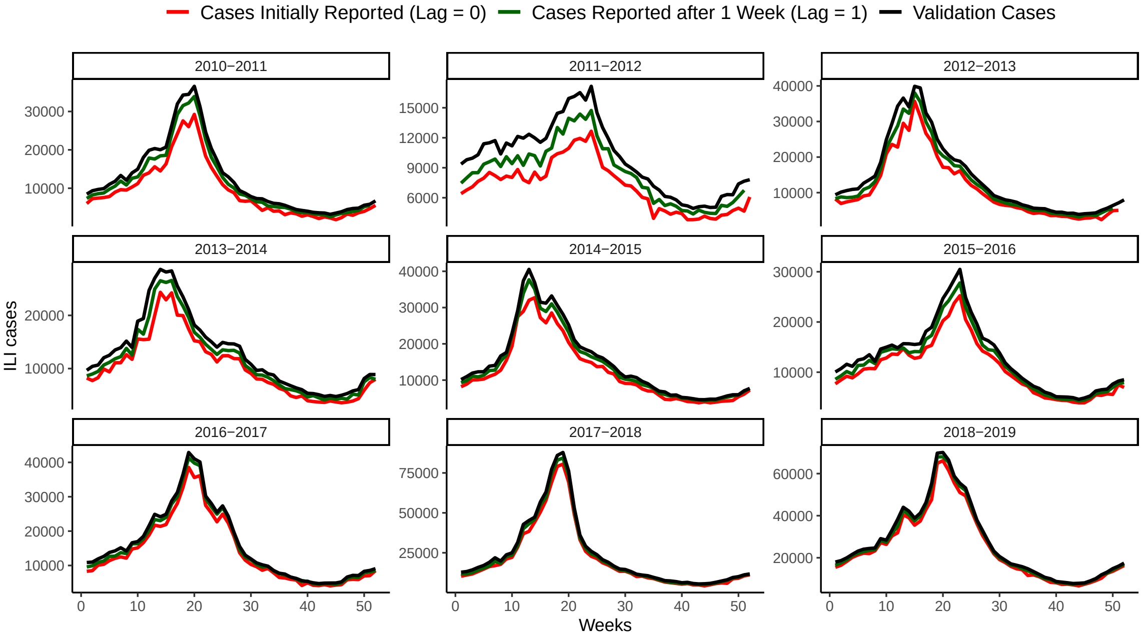

[1] In all plots, the highest (black) curve corresponds to validation case counts. The lowest (red) curve corresponds to the cases initially reported (lag = 0)

Figure B: Reported ILI cases in Vermont for each calendar week in the 2017-2018 and 2018-2019 flu seasons[1]

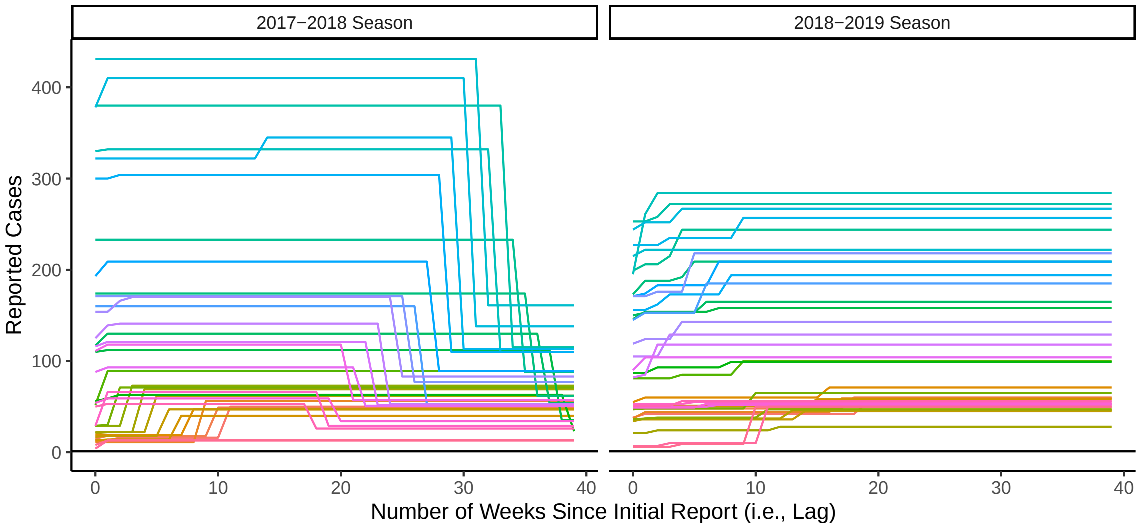

[1] Data for Vermont ILI cases were downloaded on June 13th, 2021. Lines correspond to the first 35 calendar weeks in the corresponding flu season.

Figure C: Estimated proportion of validation cases initially reported by state using data from the 2018-2019 season and data from the previous two seasons[1]

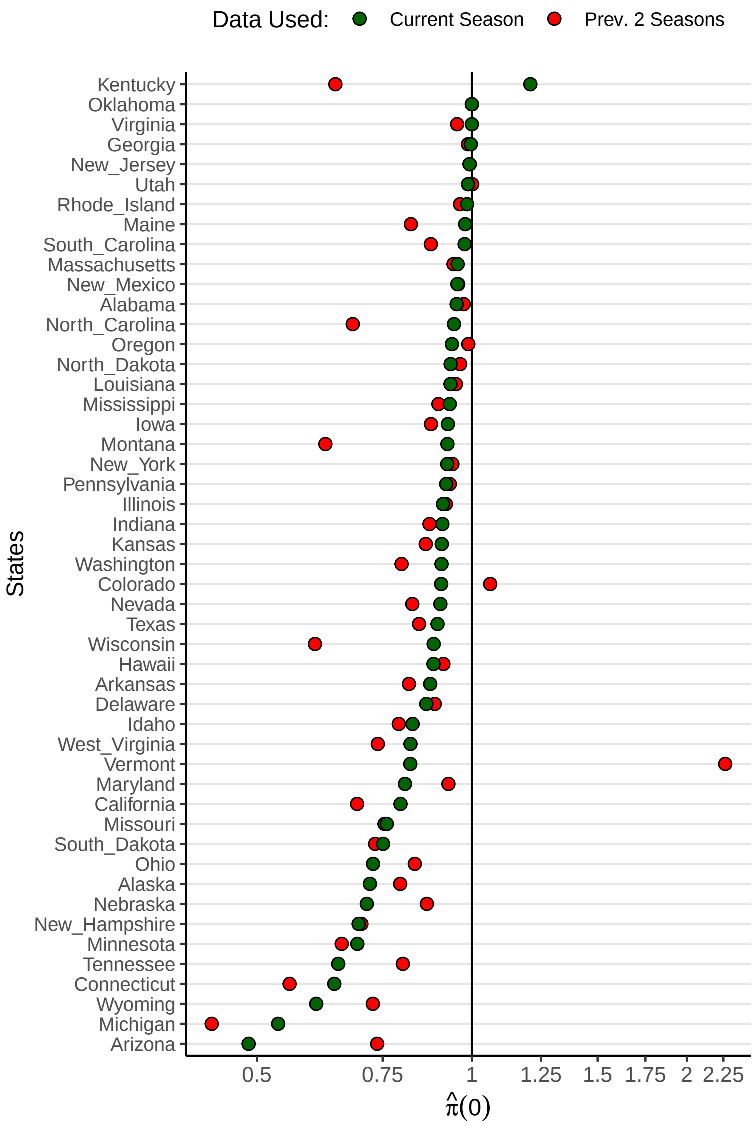

[1] Data were downloaded on June 13th, 2021. Results from the current season may be viewed as the "truth" here, but data analysis and forecasting is conducted using estimates from the previous two seasons (except for local lag-based estimation).

2 Summary of notation

Table A provides a summary of the notation used throughout the paper.

Table A: Summary of key notation

**Notation**	**Definition**
s	indexes seasons
t	indexes weeks within each season
d	indexes number of weeks since initial report for season s and week t<br>also referred to as "lag", where lag = 0 refers to the first official report
$N_{ts}(\infty)$	observed validation data for season s and week t
$N_{ts}(d)$	data reported as of the $(d+1)^{th}$ report for season s and week t<br>i.e, data from the dth data revision for season s and week t<br>$N_{ts}(0)$ corresponds to the first data report for season s and week t
$n_{ts}(d)$	$n_{ts}(d) = N_{ts}(d) - N_{ts}(d-1)$ for $d \geq 1$ and $n_{ts}(0) = N_{ts}(0)$.
$\tilde{N}_{ts}$	the most recently-reported case counts for season s and week t
$\mathbf{N}_{ts}(\infty)$	random variable corresponding to the validation data for season s and week t<br>$N_{ts}(\infty)$ is the data realization of this random variable
$\mathbf{N}_{ts}(d)$	random variable corresponding to the dth data revision for season s and week t<br>$N_{ts}(d)$ is the data realization of this random variable
$\pi_{ts}(d)$	the average proportion of validation cases that are reported by lag week d<br>$= E(\mathbf{N}_{ts}(d)/\mathbf{N}_{ts}(\infty))$
τ	value of d such that we assume $\pi_{ts}(d) = 1$ for all $d > \tau$

3 Modeling available cases with an offset

Reframing results in the actuarial literature using our notation, we assume that the *incremental* cases reported for week t in season s on lag week d follow a Poisson distribution with mean as follows:

$$E(\mathbf{n}_{ts}(d)) = y_d E(\mathbf{N}_{ts}(\infty))$$

for all $d > 1$ such that y_d represents the average number of incremental cases for lag d, relative to the expected number of validation cases, $E(\mathbf{N}_{ts}(\infty))$ [1]. Actuarial literature usually assumes that the number of reported cases is non-decreasing across lag weeks such that $y_d > 0$ for all $d \geq 0$ and $\sum_{k=0}^{\infty} y_k = 1$. We define the development factor λ_d as follows

$$\lambda_d = \frac{\sum_{k=0}^{d} y_k}{\sum_{k=0}^{d-1} y_k} = \frac{\pi(d)}{\pi(d-1)},$$

where $y_0 = \pi(0)$ and $y_d = \pi(d) - \pi(d-1)$.

It can be shown ([1]) that these assumptions imply that $\mathbf{n}_{ts}(d)|N_{ts}(d-1)$ is distributed such that

$$E(\mathbf{n}_{ts}(d)|N_{ts}(d-1)) = \frac{y_d}{\pi(d-1)} N_{ts}(d-1) = [\lambda_d - 1]\, N_{ts}(d-1)$$
$$Var(\mathbf{n}_{ts}(d)|N_{ts}(d-1)) = \frac{y_d \pi(d)}{[\pi(d-1)]^2} N_{ts}(d-1) = \lambda_d [\lambda_d - 1]\, N_{ts}(d-1).$$

We emphasize that this variance expression requires $\lambda_d > 1$ (so, $\pi(d) > \pi(d-1)$) for all $d > 1$ in order to produce sensible variance estimates. We can equivalently express the mean and variance of the cumulative cases as follows:

$$E(\mathbf{N}_{ts}(d)|N_{ts}(d-1)) = \lambda_d N_{ts}(d-1) \qquad (Eq\ a)$$
$$Var(\mathbf{N}_{ts}(d)|N_{ts}(d-1)) = \lambda_d [\lambda_d - 1]\, N_{ts}(d-1)$$

for all $d > 1$. Following England and Verrall (2002) [2], we have that

$$E(\mathbf{N}_{ts}(d)|N_{ts}(0)) = E(E(\mathbf{N}_{ts}(d)|\mathbf{N}_{ts}(d-1))|N_{ts}(0)) = \ldots = \left[\prod_{k=1}^{d} \lambda_k\right] N_{ts}(0)$$
$$Var(\mathbf{N}_{ts}(d)|N_{ts}(0)) = \prod_{k=1}^{d} \lambda_k \left[\prod_{k=1}^{d} \lambda_k - 1\right] N_{ts}(0).$$

Using that $\mathbf{N}_{ts}(0)$ has a Poisson distribution with mean $y_0 E(\mathbf{N}_{ts}(\infty))$, we then have that $E(\mathbf{N}_{ts}(d)) = \pi(d) E(\mathbf{N}_{ts}(\infty))$ and that

$$\begin{aligned} Var(\mathbf{N}_{ts}(d)) &= E(Var(\mathbf{N}_{ts}(d)|\mathbf{N}_{ts}(0))) + Var(E(\mathbf{N}_{ts}(d)|\mathbf{N}_{ts}(0))) \\ &= \prod_{k=1}^{d} \lambda_k \left[\prod_{k=1}^{d} \lambda_k - 1\right] E(\mathbf{N}_{ts}(0)) + \left[\prod_{k=1}^{d} \lambda_k\right]^2 Var(\mathbf{N}_{ts}(0)) \\ &= \pi(d) \left[\frac{\pi(d)}{\pi(0)} - 1\right] E(\mathbf{N}_{ts}(\infty)) + \frac{\pi(d)^2}{\pi(0)} E(\mathbf{N}_{ts}(\infty)) \\ &= \pi(d) E(\mathbf{N}_{ts}(\infty)) \left[2 \left[\frac{\pi(d)}{\pi(0)}\right] - 1\right]. \qquad (Eq\ b) \end{aligned}$$

All of this is to say that $\mathbf{N}_{ts}(d)$ will follow a distribution with mean $\pi(d)E(\mathbf{N}_{ts}(\infty))$ and variance proportional to $\pi(d)E(\mathbf{N}_{ts}(\infty))$. It may reasonable, therefore, to approximate the distribution of $\mathbf{N}_{ts}(d)$ using an over-dispersed Poisson or negative binomial distribution with mean $E(\mathbf{N}_{ts}(d)) = \pi(d)E(\mathbf{N}_{ts}(\infty))$.

Suppose that we are interested in modeling the validation case counts $\mathbf{N}_{ts}(\infty)$ using a model

structure with a log link. Then this implies the following mean model structure for the available counts

$$\log(E(\mathbf{N}_{ts}(d)) = \log(E(\mathbf{N}_{ts}(\infty))) + \log(\pi(d)). \qquad (Eq\ c)$$

This follows the same mean structure as the model for the validation case counts but includes an offset, $\log(\pi(d))$. This mean model structure and modeling strategy are very closely related to the approach used in McGough et al. (2020) [3], which modeled incremental cases rather than cumulative cases as follows:

$$\log(E(\mathbf{n}_{ts}(d)) = \log(E(\mathbf{N}_{ts}(\infty))) + \log(y_d).$$

The above model in McGough et al. (2020) [3] requires that $y_d > 0$ for all d. However, the model structure in *Eq c* can be applied to model cumulative cases as long as $\pi(d) > 0$ for all d, albeit without the variance structure justification in *Eq b*. This greatly expands the scenarios in which the model structure in *Eq c* can be applied, allowing the method to be implemented in settings with over-reporting of cases (i.e., $\lambda_d < 1$ for some d) in addition to under-reporting.

Alternative modeling strategies discussed in the actuarial literature [e.g. 2;4] involve approximating the distribution of $\mathbf{N}_{ts}(d)|N_{ts}(d-1)$ with a normal distribution with mean and variance as in *Eq a* or assuming some other distributional approximation such as a log-normal distribution. In the main paper, we consider ARMA modeling of log-cumulative case counts, where the mean structure of the model follows *Eq c*.

4 Obtaining multiple imputations for validation case counts

4.1 Multiple imputation prior to disease modeling

One strategy for handling reporting delay proposed in the main paper involves imputing missing validation case counts given the observed data. In this section, we provide some motivation for the distributional assumptions made for this imputation. As discussed in England and Verrall (2002) [2] and following *Eq a*, we can express the moments of the distribution for $\mathbf{N}_{ts}(\infty)|N_{ts}(d)$ as:

$$E(\mathbf{N}_{ts}(\infty)|N_{ts}(d)) = \prod_{k=d+1}^{\infty} \lambda_k N_{ts}(d) = \frac{N_{ts}(d)}{\pi(d)}$$

$$Var(\mathbf{N}_{ts}(\infty)|N_{ts}(d)) = \left[\frac{1}{\pi(d)}\right]\left[\frac{1-\pi(d)}{\pi(d)}\right] N_{ts}(d) = \left[\frac{1-\pi(d)}{\pi(d)}\right] E(\mathbf{N}_{ts}(\infty)|N_{ts}(d))$$

in the setting where $\pi(d) \leq 1$ for all d (i.e., reporting error is from under-reporting). Given an estimate of $E(\mathbf{N}_{ts}(\infty)|N_{ts}(d))$ and allowing for a more general reporting delay mechanism that may vary by s and/or t, we propose approximating the distribution of $\mathbf{N}_{ts}(\infty)$ using the following truncated normal distribution:

$$\mathbf{N}_{ts}(\infty)|N_{ts}(d) \sim TruncNormal\left(\frac{N_{ts}(d)}{\pi_{ts}(d)}, \frac{1-\pi_{ts}(d)}{\pi_{ts}(d)^2}N_{ts}(d); l, u\right), \qquad (Eq\ d)$$

where truncation limits $l = N_{ts}(d)$ and $u = \infty$ restrict imputed values to be greater than $N_{ts}(d)$. The key to this imputation distribution is that it is centered near the expected validation value and its variability generally decreases as $\pi_{ts}(d)$ increases. Therefore, the variability of the imputations decreases as the expected proportion of eventually reported cases being currently reported increases. To implement imputation using *Eq d*, we will replace unknown $\pi_{ts}(d)$ with an estimate. This results in so-called "improper" imputations that do not account for uncertainty in the estimation of $\pi_{ts}(d)$ [5]. However, we do not expect this to result in much loss of forecast coverage in practice unless the amount of estimation error for $\pi_{ts}(d)$ is very large.

As an aside, we note that the expectation of $\mathbf{N}_{ts}(\infty)|N_{ts}(d)$ from a truncated normal distribution will not be $\frac{N_{ts}(d)}{\pi_{ts}(d)}$. In **Figure E**, we show the percent bias between the expectation of the trucated normal distribution in *Eq d* and $\frac{N_{ts}(d)}{\pi_{ts}(d)}$ as a function of the observed case counts $N_{ts}(d)$ and $\pi_{ts}(d)$. We expect this bias to be generally small, but we could modify the center of the truncated normal distribution to have expectation exactly equal to $\frac{N_{ts}(d)}{\pi_{ts}(d)}$.

When we have that $\pi_{ts}(d) > 1$, we propose the following modified normal distribution, which has a variance structure with similar properties as in *Eq d*:

$$\mathbf{N}_{ts}(\infty)|N_{ts}(d) \sim Binomial\left(N_{ts}(d), \frac{1}{\pi_{ts}(d)}\right)$$

$$\text{or} \sim TruncNormal\left(\frac{N_{ts}(d)}{\pi_{ts}(d)}, \frac{\pi_{ts}(d)-1}{\pi_{ts}(d)^2}N_{ts}(d); l, u\right),$$

where truncation limits $l = -\infty$ and $u = N_{ts}(d)$ restrict imputed values to be less than $N_{ts}(d)$.

Using one of the above imputation distributions, we generate M versions of the complete corrected validation data as shown in **Figure D**. We then fit the disease model of interest to each of the M complete validation datasets and obtain M forecasts (e.g., 1-week forecasts), denoted $\hat{\mu}_1, \ldots, \hat{\mu}_M$. Let $v_1, \ldots, v_M$ represent the corresponding variance estimates for these forecasts. We can obtain a single forecast estimate and corresponding variance using Rubin's combining rules [5] as follows:

$$\hat{\mu} = \frac{1}{M}\sum_{m=1}^{M}\hat{\mu}_m \qquad \text{Var}(\hat{\mu}) = V_W + \left(1 + \frac{1}{M}\right)V_B$$

$$\text{where } V_W = \frac{1}{M}\sum_{m=1}^{M} v_m \text{ and } V_B = \frac{1}{M-1}\sum_{m=1}^{M}\left[\hat{\mu}_m - \hat{\mu}\right]^2.$$

In this expression, V_W represents the average forecast variance *within* each imputed dataset and V_B captures the variability in forecasts *across* imputed datasets.

Figure D: Diagram of Multiple Imputation Algorithm

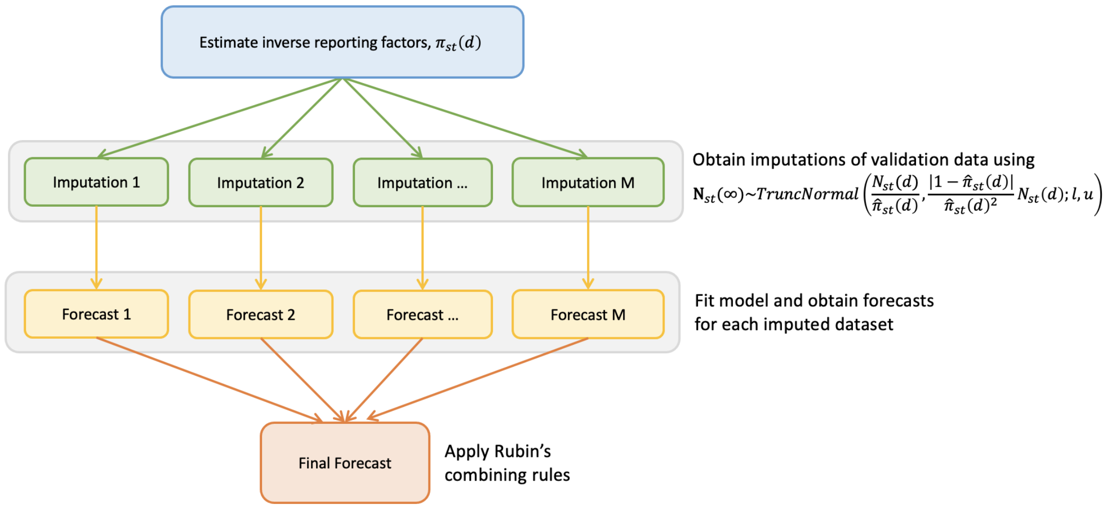

Figure E: Percent error between truncated normal expectation and $E(\mathbf{N}_{ts}(\infty)|N_{ts}(d))$

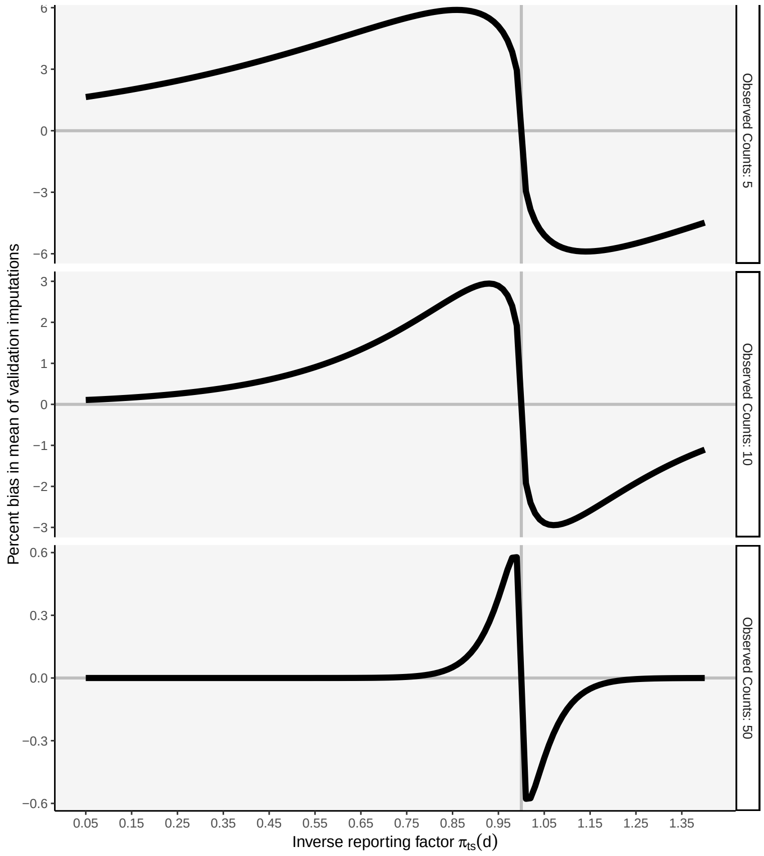

4.2 Imputation within MCMC algorithm for Bayesian forecast models

A primary limitation of the above approach is that it requires the forecast model to be fit multiple times. For many slow estimation methods, this may not be feasible. When the target model involves Markov Chain Monte Carlo (MCMC) estimation, however, a simple alternative is to handle the missing data within the estimation algorithm, where a single imputed value for each missing $N_{ts}(\infty)$ is generated for each iteration of the MCMC algorithm. Parameters are then drawn within that iteration conditioning on the imputed validation data. The resulting posterior forecast distributions can then be used directly.

In a joint modeling framework, the distribution used to impute $\mathbf{N}_{ts}(\infty)$ is calculated using the combination of the forecast model (distribution of $\mathbf{N}_{ts}(\infty)$) and a model relating $N_{ts}(d)$ to $N_{ts}(\infty)$. For this second component, we model the distribution for $\mathbf{N}_{ts}(d)|N_{ts}(\infty)$. Using similar logic as in *Eq d* above and assuming that $\pi_{ts}(d) \leq 1$, we can approximate this distribution as follows:

$$\begin{aligned}\mathbf{N}_{ts}(d)|N_{ts}(\infty) &\sim TruncNormal\left(\pi_{ts}(d)N_{ts}(\infty), [1-\pi_{ts}(d)]\pi_{ts}(d)N_{ts}(\infty); l, u\right) \qquad (Eq\ e)\\ &\text{or} \sim Binomial(N_{ts}(\infty), \pi_{ts}(d)),\end{aligned}$$

where $l = 0$ and $u = N_{ts}(\infty)$ and where the second expression is based on the relationship between Binomial and Normal distributions and assumes that $0 < \pi_{ts}(d) \leq 1$ for all d (i.e., no over-reporting of disease). This second distribution is also used in Hohle et al. (2014) [6]. In the setting where $\pi_{ts}(d) > 1$, we propose modeling the observed data as follows:

$$\mathbf{N}_{ts}(d)|N_{ts}(\infty) \sim TruncNormal\left(\pi_{ts}(d)N_{ts}(\infty), [\pi_{ts}(d)-1]\pi_{ts}(d)N_{ts}(\infty); l, u\right), \qquad (Eq\ f)$$

where $l = N_{ts}(\infty)$ and $u = \infty$.

5 Forecasting models

In this section, we describe the two forecasting models we use in the main paper. Crucially, the reporting delay methods can be applied to many forecasting model structures beyond those explored here.

5.1 Inferno Gaussian process model for case counts

Osthus (2020) [7] proposes a Bayesian strategy for modeling weighted influenza-like illness (wILI) rates (scaled and population-weighted case counts) reported by ILInet. This approach results in substantially faster computation relative to usual Bayesian modeling of these data due to the pre-estimation of many model parameters prior to Bayesian Markov Chain Monte Carlo (MCMC) estimation.

Let $\tilde{\mathbf{w}}_{ts}$ denote a random variable with the most recently-reported wILI value for week t in season s as its data realization. The model is as follows:

$$
\begin{aligned}
&\tilde{\mathbf{w}}_{ts}|\alpha, \theta_{ts} \sim Beta(\alpha\theta_{ts}, \alpha(1-\theta_{ts}))\\
&\text{logit}(\theta_{ts}) = \gamma_t + \delta_{ts}\\
&\delta_{ts}|\mu_s, \Sigma \sim GP(\mu_s\mathbf{1}, \Sigma)\\
&\mu_s|\sigma_\mu^2 \sim N(0, \sigma_\mu^2)\\
&\Sigma_{i,i} = \sigma_\Sigma^2\\
&\Sigma_{i,j\neq i} = \phi\sigma_\Sigma^2 \exp -\lambda(i-j)^2
\end{aligned}
$$

where GP denotes a Gaussian process. Parameters λ, α, σ_μ^2, and σ_Σ^2 are all scalar parameters greater than 0, and ϕ is a scalar parameter between 0 and 1. If we also specify a prior distribution for γ_t, this model could be fit directly to the observed data. However, the large number of parameters in each γ_t and δ_{ts} may make estimation slow. Instead, Osthus (2020) [7] proposes to pre-estimate some of the model parameters (α, γ, σ_μ^2, σ_Σ^2, λ, ϕ, and μ_s and δ_{ts} for past seasons) based on past season historical data and only estimate a small number of parameters using the current season's data. This substantially reduces the computational burden of estimation, since we are then only estimating μ_s and δ_{ts} using data from the current season.

This model is intended to be applied for wILI $\tilde{y}_{ts}$ between 0 and 1. We adapt this model structure for the context where the outcome of interest is the raw case counts rather than a scaled and weighted version. In particular, we propose the following model structure:

$$
\begin{aligned}
&\tilde{\mathbf{N}}_{ts}|\alpha, \theta_{ts} \sim NegBin(\alpha, \alpha/(\alpha+\theta_{ts}))\\
&\log(\theta_{ts}) = \gamma_t + \delta_{ts}\\
&E(\tilde{\mathbf{N}}_{ts}|\alpha, \theta_{ts}) = \theta_{ts} \qquad Var(\tilde{\mathbf{N}}_{ts}|\alpha, \theta_{ts}) = \frac{\theta_{ts}(\theta_{ts}+\alpha)}{\alpha}
\end{aligned}
$$

where $\tilde{\mathbf{N}}_{ts}$ is a random variable with the most recent case count for week t and season s as its data realization. We define distributions of all hyperparameters as in Osthus (2020) [7]. The key changes between the proposed model and the model in Osthus (2020) [7] is in the assumed distribution for the observed outcome and in the link function used to model the mean of the outcome. The parameter pre-estimation step also needs to be modified accordingly.

Here, we describe how we obtain pre-estimates for the various (modified) Inferno model parameters using historical data. For this estimation, we only consider the historical *validation* data. We perform the following estimation steps:

1. Estimate θ: Define $\hat{\beta}_{ts}$ to be a three-week moving average of the validated counts, $N_{ts}(\infty)$ for s corresponding to *past* seasons. Let $\hat{\omega}_t$ be the average of $N_{ts}(\infty)/\hat{\beta}_{ts}$ across prior seasons.

Define $\hat{\theta}_{ts} = \max\left(\hat{\beta}_{ts}\hat{\omega}_t, c\right)$ where c is some pre-specified minimum value. In our data analysis and simulations, we use $c = 0.005$.

2. Estimate α: We assume $\tilde{\mathbf{N}}_{ts}$ follows a negative binomial distribution with mean $\hat{\theta}_{ts}$ and unknown parameter α. Using the historical validation data, we maximum the log-likelihood corresponding to the negative binomial distribution as a function of α to obtain an estimate of α.

3. Estimate γ_t and δ_{ts}: We estimate $\hat{\gamma}_t$ as the average of $\log(\hat{\theta}_{ts})$ across s. We obtain $\hat{\delta}_{ts} = \log(\hat{\theta}_{ts}) - \hat{\gamma}_t$.

4. Estimate σ^2_μ: We estimate $\hat{\mu}_s$ as the mean of $\hat{\delta}_{ts}$ for each season. $\hat{\sigma}^2_\mu$ is the estimated variance of $\hat{\mu}_s$ across seasons.

5. Estimate Σ: We estimate $\hat{\sigma}^2_\Sigma$ as the variance of δ_{ts} across all t and s. We assume that vector $\delta_s|\mu_s$ follows a multivariate normal distribution with mean $\hat{\mu}_s$ and variance Σ, which is a function of parameters λ and ϕ. Using the estimated $\hat{\delta}_{ts}$, we maximize the multivariate normal log-likelihood as a function of λ and ϕ to obtain corresponding estimates. Combined, this provides an estimate for Σ.

Given these pre-estimates, we can then apply a Bayesian MCMC algorithm to estimate the remaining parameters (δ_{ts}) corresponding to the *current* season. When accounting for reporting delay using the rescaling method, we fit this model on a rescaled version of the current season data, and this rescaling would occur prior to MCMC estimation. When implementing the mean model offset method, we modify the above mean model to $\log(\theta_{ts}) = \gamma_t + \delta_{ts} + \log(\hat{\pi}_{ts}(d))$ where $\log(\hat{\pi}_{ts}(d))$ is a fixed offset. Parameter pre-estimation is not impacted. To implement the imputation strategy, we can add a step within each iteration of the MCMC estimation algorithm in which we impute the validation data. Inferno model parameters are then drawn, conditional on the imputed validation data. In this paper, we implement the MCMC estimation using the *rjags* package in R and instead specify a model for $N_{ts}(d)|N_{ts}(\infty)$ as in *Eq e* or *Eq f*.

5.2 ARMA model for log-case counts

We also model the data using an ARMA(p,q) model as follows:

$$x_{ts} = c + \sum_{i=1}^{p} \phi_i x_{t-i,s} + \sum_{i=1}^{q} \theta_i \epsilon_{t-i,s} + \epsilon_{ts}, \qquad \epsilon_{ts} \sim N(0, \sigma^2), \qquad (Eq\ g)$$

where x_{ts} is the log of the most recently-reported case data for week t and season s and where $\epsilon_{t-i,s} = x_{t-i,s} - x_{t-i-1,s}$ is the increment in log-counts for week $t-i$. In our implementation, we added 0.1 to each case count to avoid log-of-zero problems. When implementing the mean model offset method, we add $\log(\hat{\pi}_{ts}(d))$ as an offset in the mean structure above. The imputation method is implemented by fitting the ARMA model and obtaining forecasts for each of 10 imputed datasets. Results are then aggregated across multiple imputations as described in **Section 4**. ARMA models are usually fit using maximum likelihood or least squares methods and can be easily implemented with most modeling software.

6 Application to dengue fever and ILI data

6.1 Regression-based reporting factor estimation

In this section, we describe how we used a regression model to indirectly estimate the reporting factors and provide diagnostics assessing the reasonableness of this model.

We posit a Poisson regression model structure for $\mathbf{N}_{ts}(\infty)$ with a log link as follows:

$$\begin{aligned}\log(\theta_{ts}) = &\beta_0 + \beta_1 spline_1 + \beta_2 spline_2 + \beta_3 spline_3 \\ &+ \beta_4\mathcal{I}(d=0) + \beta_5\mathcal{I}(d=1) + \beta_6\mathcal{I}(d=2) + \beta_7\mathcal{I}(d=3) + \beta_8\mathcal{I}(d=4) + \beta_9\mathcal{I}(d=5) \\ &+ \beta_{10}s + \log\left(N_{ts}(d) + 0.001\right)\end{aligned} \qquad (Eq\ h)$$

where θ_{ts} is the expectation of $\mathbf{N}_{ts}(\infty)$ given the predictors used in the model, where $spline_1$, $spline_2$, and $spline_3$ collectively represent a 3-degree natural spline of t, and where the 0.001 was added in the offset to avoid log-of-zero errors. We note that these β parameters are defined differently than in **Supp. Section 5.1**. For each calendar week, we estimated parameters in this model using the data on $N_{ts}(d)$ and $N_{ts}(\infty)$ for all t and s in the previous two years and all d available, excluding data from the last 6 weeks (dengue fever and simulations) or last 16 weeks (influenza-like illness).

We decided to include s as a continuous variable in this model to allow for seasonal trends such as improved reporting for more recent seasons. While this model is fit using the past two years of data, this will included data from at least two seasons, allowing the corresponding parameter to be estimated. A continuous rather than a categorical version of s is used in the model to improve current season predictions early in the current season, where the model would be fit only using past season data and no current season data.

For simplicity, we evaluate the goodness-of-fit of this model using the entire time series of available data for each disease. **Figure F** shows the regression model predictions for $\pi_{ts}(d)$ compared to the observed ratio between $N_{ts}(d)$ and $N_{ts}(\infty)$. Note that this is evaluating these estimates in the model's training set. For influenza-like illness, we see that the model is able to do a pretty good job of estimating these inverse reporting factors in general. For the dengue fever data, the model has a harder time determining the values for $\pi_{ts}(1)$, the second report produced for week t in season s. For other lag weeks, however, the model does a reasonable job at recovering the true inverse reporting factors.

Figure F: Comparison of regression model *estimates* of $\pi_{ts}(d)$ from *Eq. 7* based on historical real-time case reporting and the *observed values* of $\pi_{ts}(d) = N_{ts}(d)/N_{ts}(\infty)$ for national US ILI and for dengue fever in Puerto Rico.[1]

(a) US national influenza-like illness

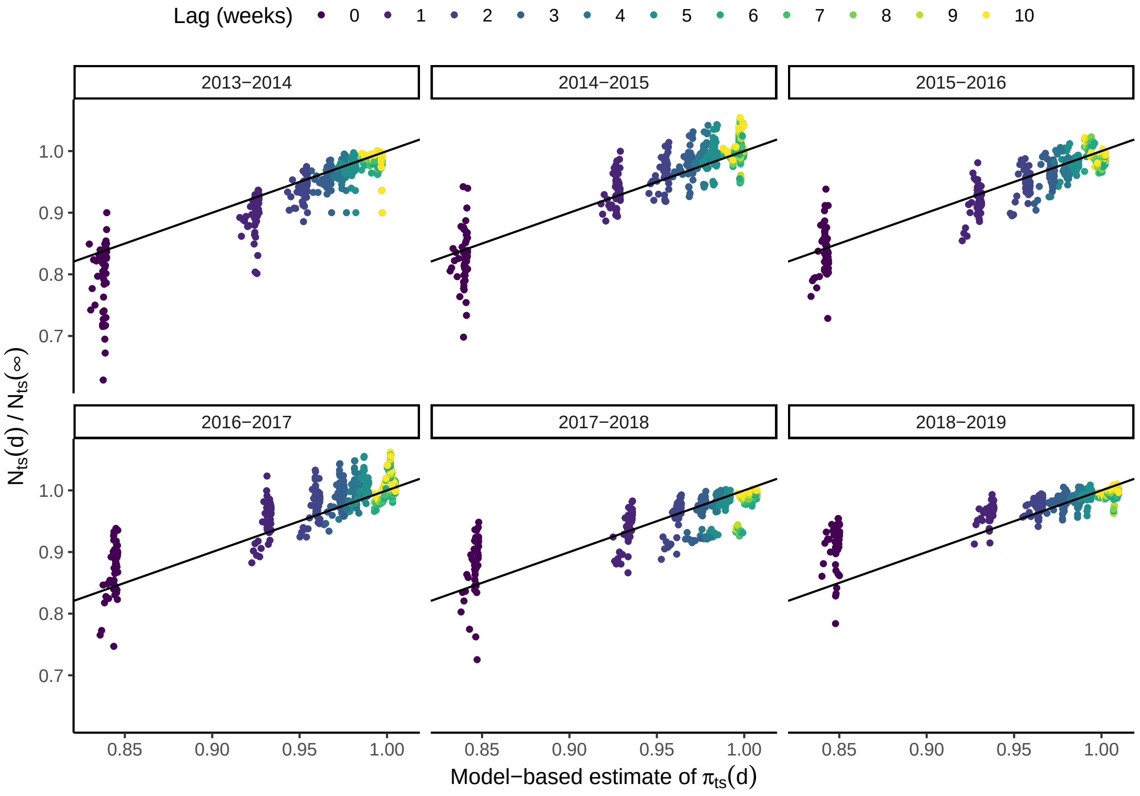

(b) Dengue fever in Puerto Rico

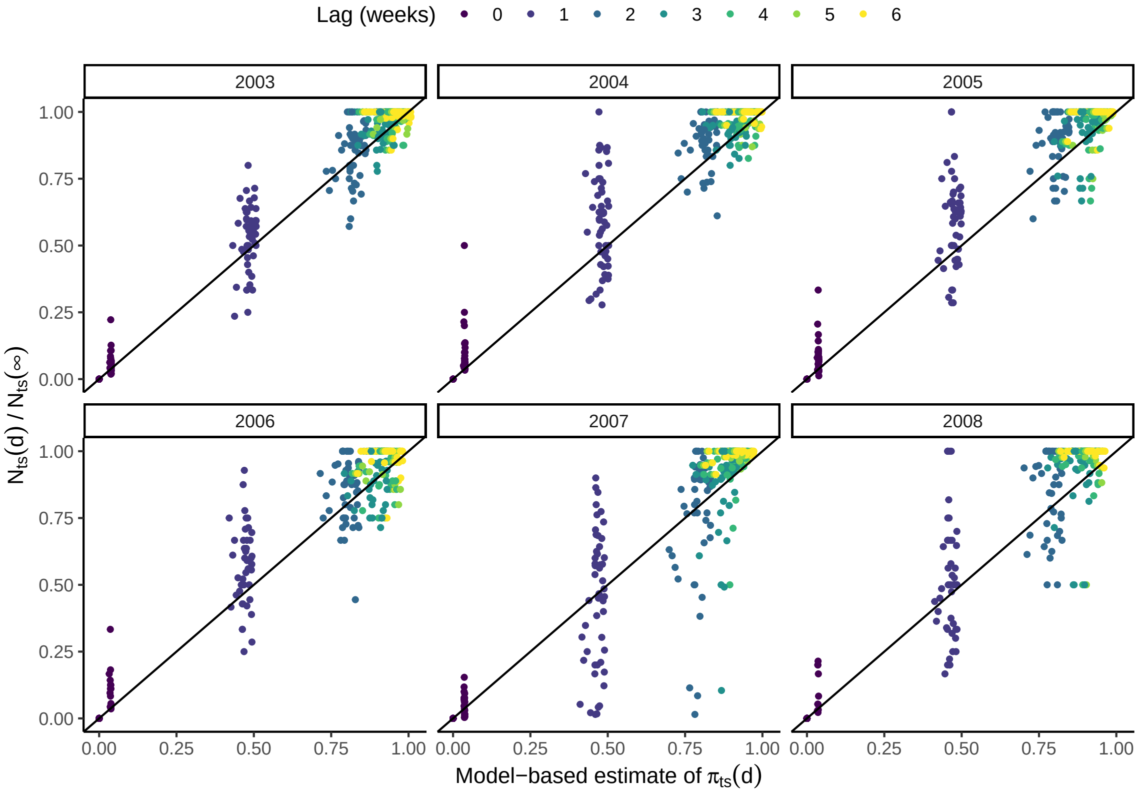

[1] Model-based estimates of $\pi_{ts}(d)$ are obtained by fitting the regression model in *Eq h* to historical reporting data, excluding the most recent 16 weeks (ILI) or the most recent 6 weeks (dengue fever) from the estimation. Values along the black line indicate model-based estimates of $\pi_{ts}(d)$ that closely align with observed values of $\pi_{ts}(d)$.

6.2 Nowcast and forecast performance

In this section, we provide some additional results on forecast performance from the data analysis. **Figure G** provides aggregate method performance in terms of forecast weighted interval scores. **Figure H** provides boxplots of the nowcast and forecast errors across all weeks and seasons for each dataset. This figure is useful for comparing the presence of large error outliers for each of the methods. **Figures I and J** provide a visualization of the forecast accuracy across individual weeks for each dataset, where a larger filled portion of the vertical axis corresponds to greater accuracy (i.e., lower forecast error) for that method, relative to the other methods. These figures demonstrate that the performance of simple analysis of the observed data without correction tends to improve when case counts are very low for dengue fever. We also see improvements in the relative performance of uncorrected analysis in the US national ILI data just after peaks in the validation cases.

Figure O shows the comparative accuracy between modeling based on real-time data and modeling based on validation data in terms of 1 week forecasts. Surprisingly, Inferno modeling based on real-time data does a better job at forecasting 1 week ahead than Inferno based on validation data in the setting where the true number of validation cases is lower than expected. In 2000, for example, the season peak was later than in prior seasons, and Inferno based on validation data tended to over-forecast the case counts in the coming weeks. In contrast, Inferno based on real-time data is biased toward zero due to reporting delay, which resulted in better 1 week forecasts in the setting where validation case counts were unexpectedly low.

Figure L provides the relative rankings of the various methods for forecasting state-level ILI levels in the 2018-2019 season. Accuracy for individual weeks are plotted for several states in **Figure K**.

Figure G: Weighted interval scores of forecasts in the Puerto Rico dengue fever and national US influenza-like illness data across all weeks[1]

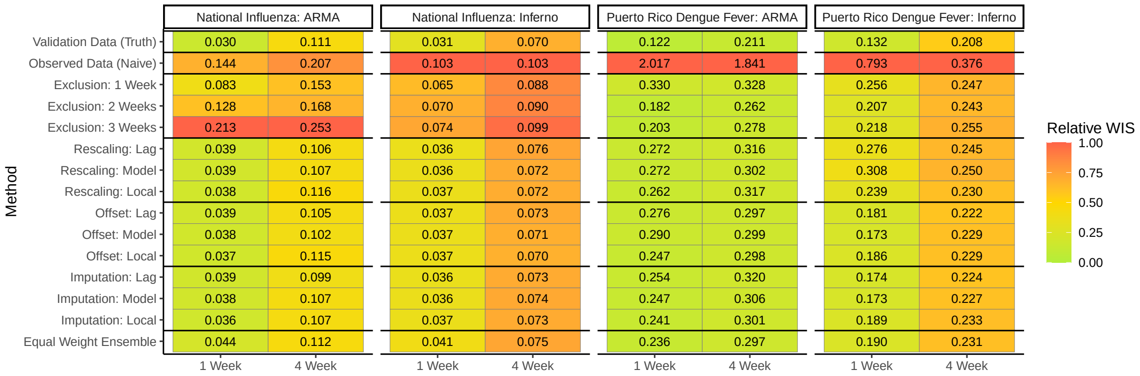

[1] Results for dengue fever are aggregated across each of 50 weeks in 18 seasons (1992-2009). Results for US national influenza are aggregated across 35 weeks in 8 seasons (2012-2018). The ensemble method corresponds to an equal-weight linear combination of all methods except validation data analysis. Relative weighted interval scores (WIS) are calculated relative to the largest value in each column.

Figure H: Boxplots of performance metrics across all weeks

(a) Season peak-scaled prediction errors for nowcasts and 1 week forecasts

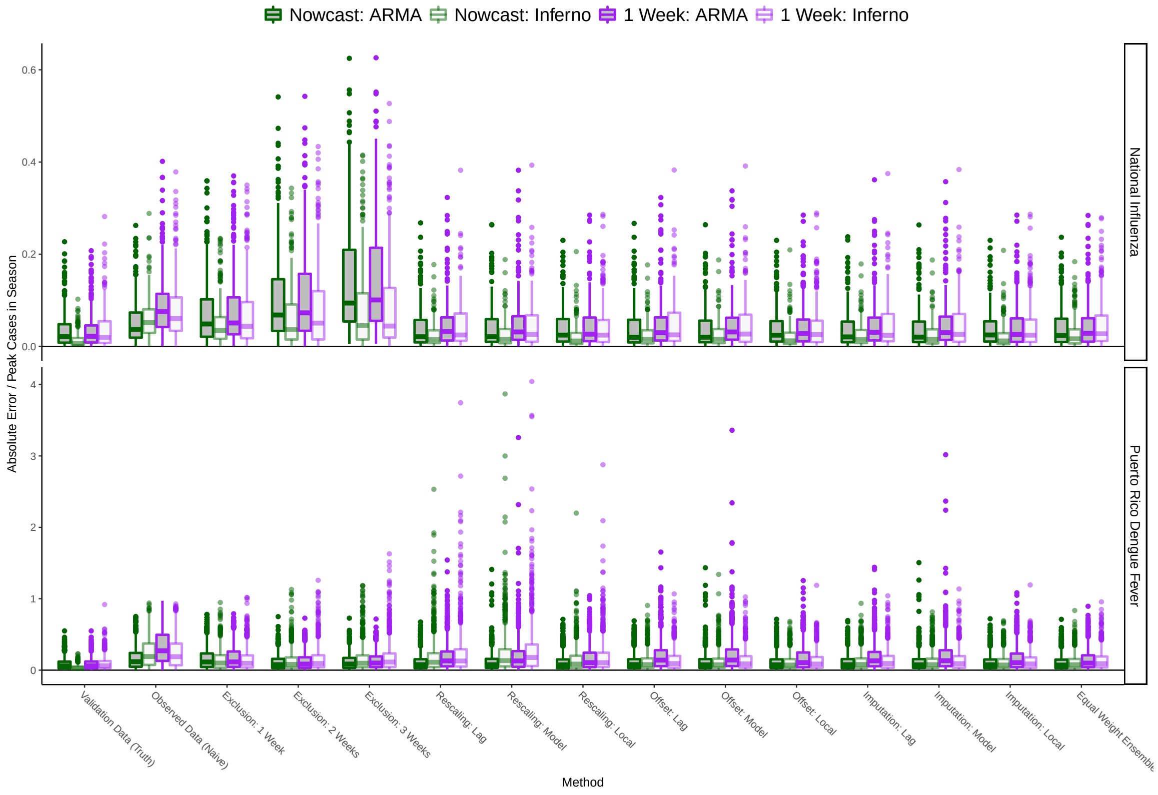

(b) Weighted interval scores for 1 and 4 week forecasts

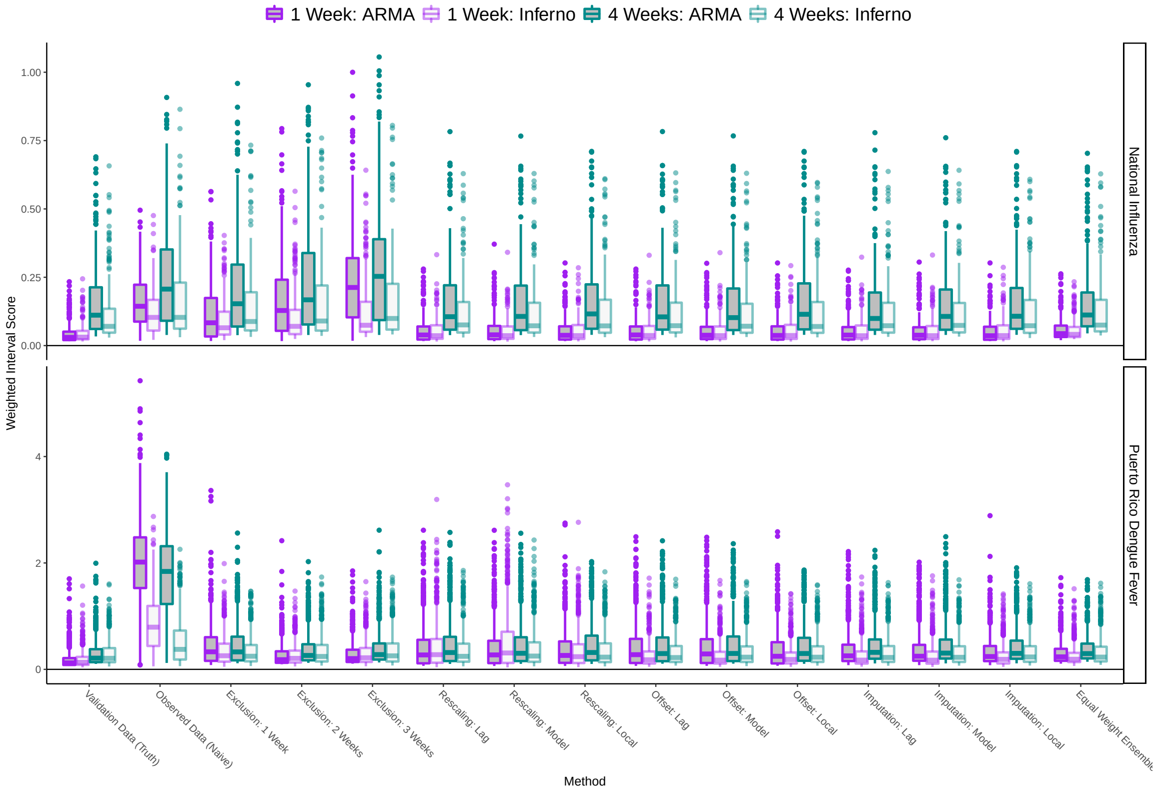

Figure I: Relative accuracy (1/absolute prediction error, scaled across methods) of 1 week ahead forecasts across rolling 5 week window for US National ILI[1]

(a) ARMA model

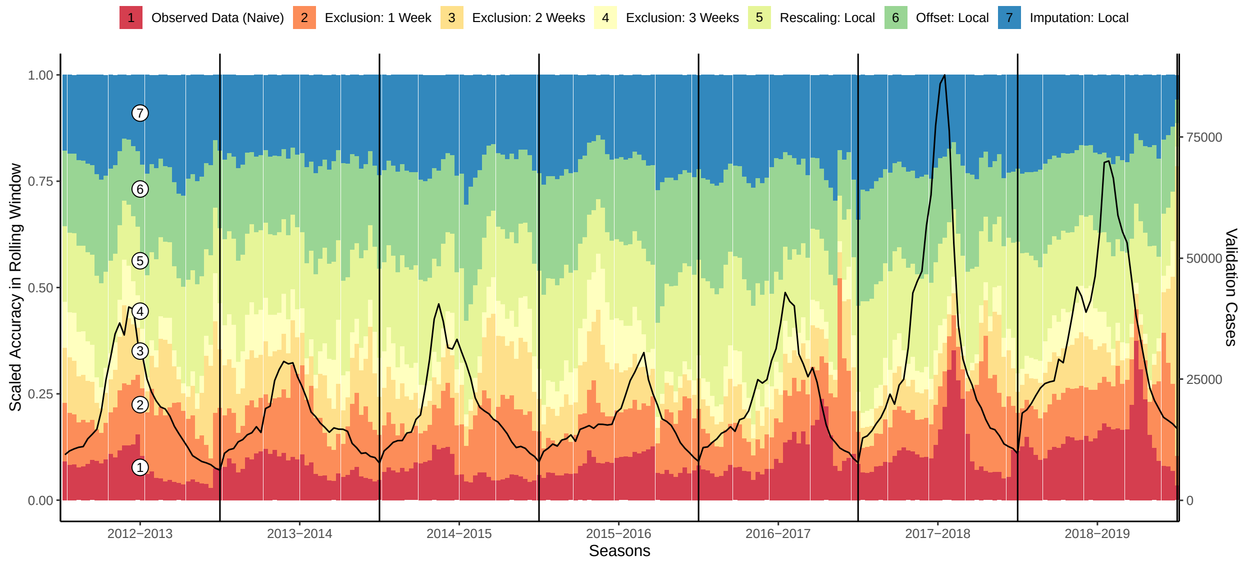

(b) Inferno model

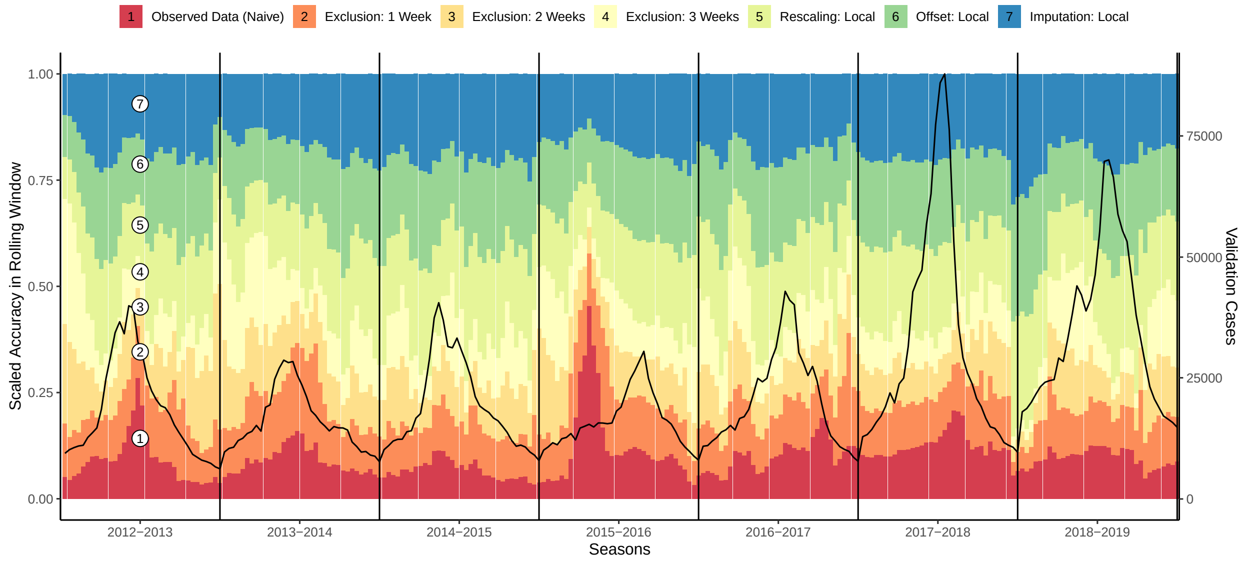

[1] Results based on absolute prediction error for 5 week rolling window centered at plotted week. Results for 35 weeks per season are shown. The black line represents observed validation case counts for each week.

Figure J: Relative accuracy (1/absolute prediction error, scaled across methods) of 1 week ahead forecasts across rolling 5 week window for Puerto Rico dengue fever[1]

(a) ARMA model

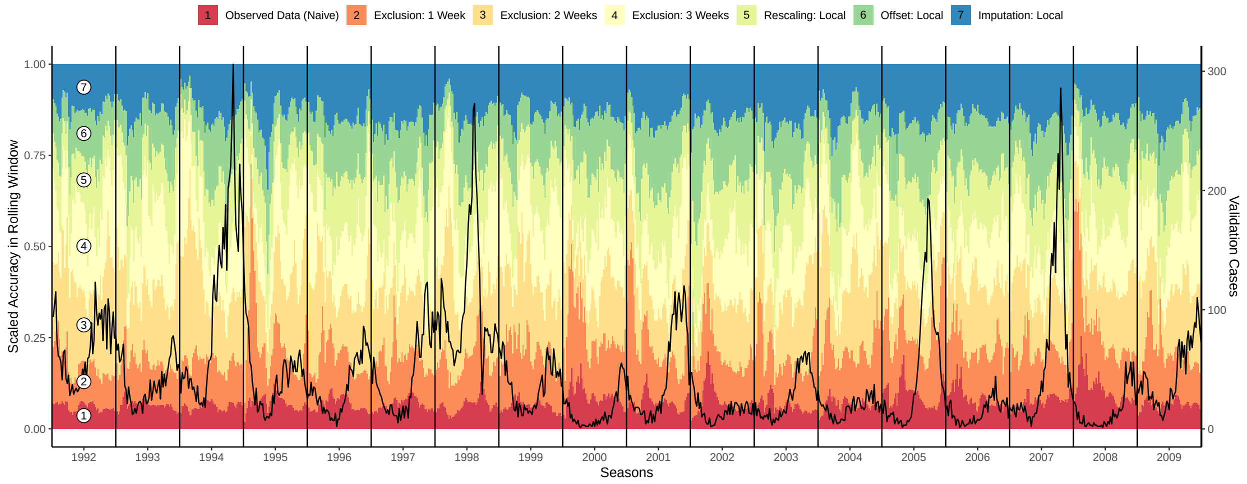

(b) Inferno model

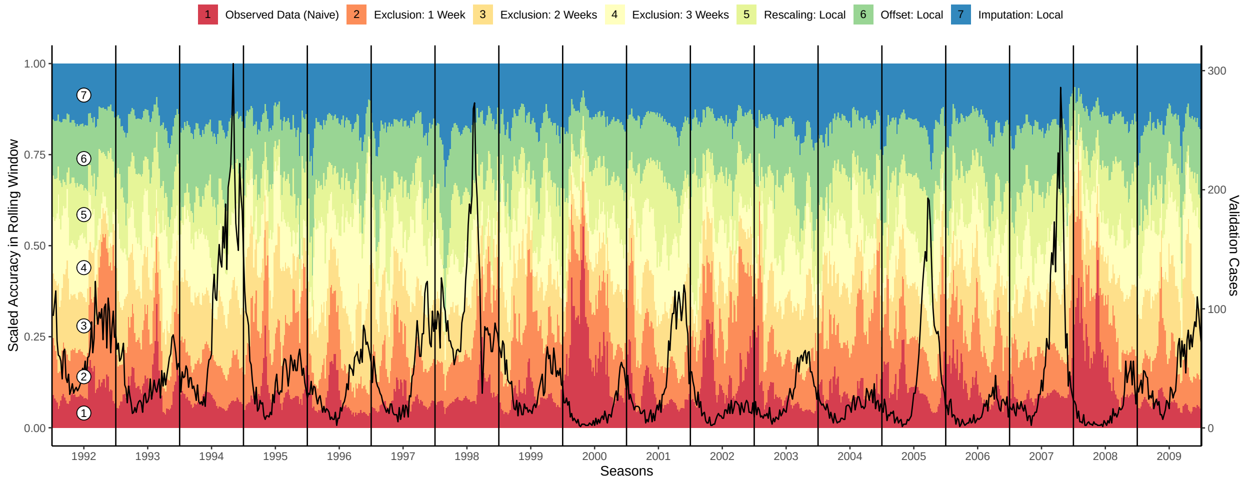

[1] Results based on absolute prediction error for 5 week rolling window centered at plotted week. Results for 50 weeks per season are shown. The black line represents observed validation case counts for each week.

Figure K: Relative accuracy (1/absolute prediction error, scaled across methods) of 1 week ahead forecasts across rolling 5 week window for state-level ILI in 2018-2019 season[1]

(a) ARMA model

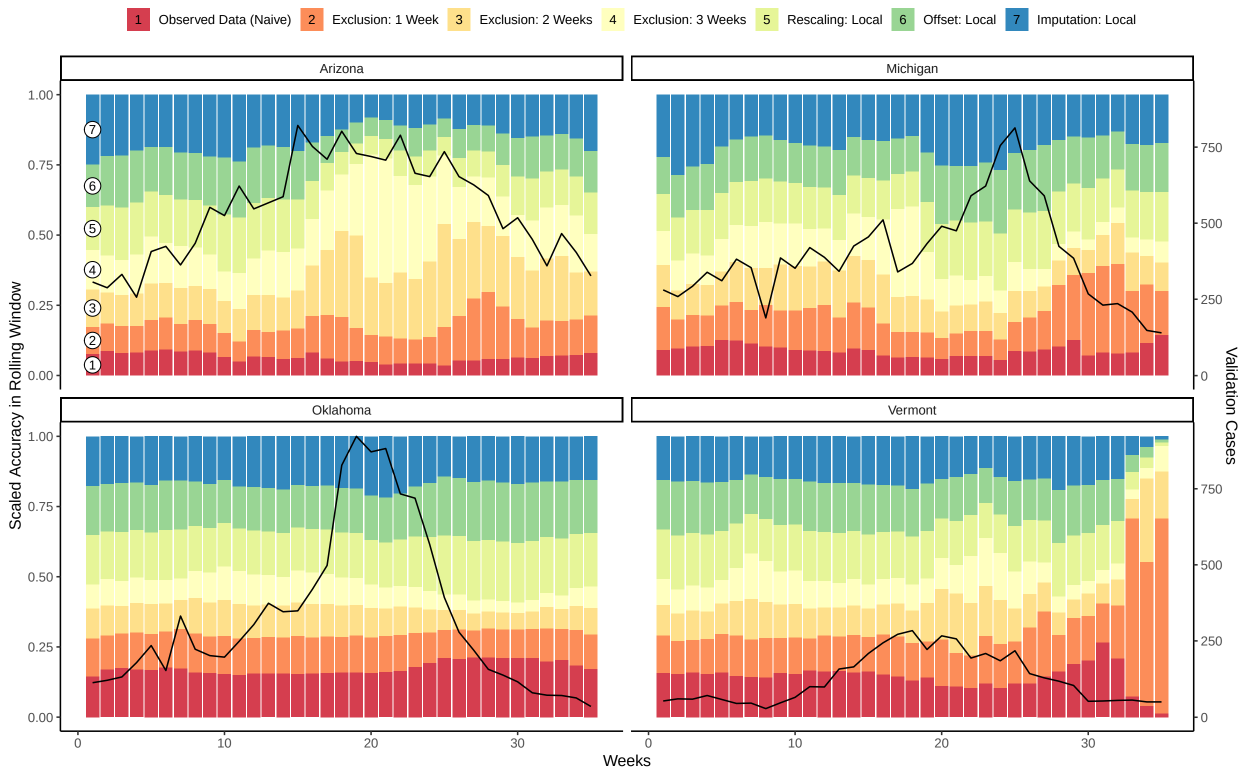

(b) Inferno model

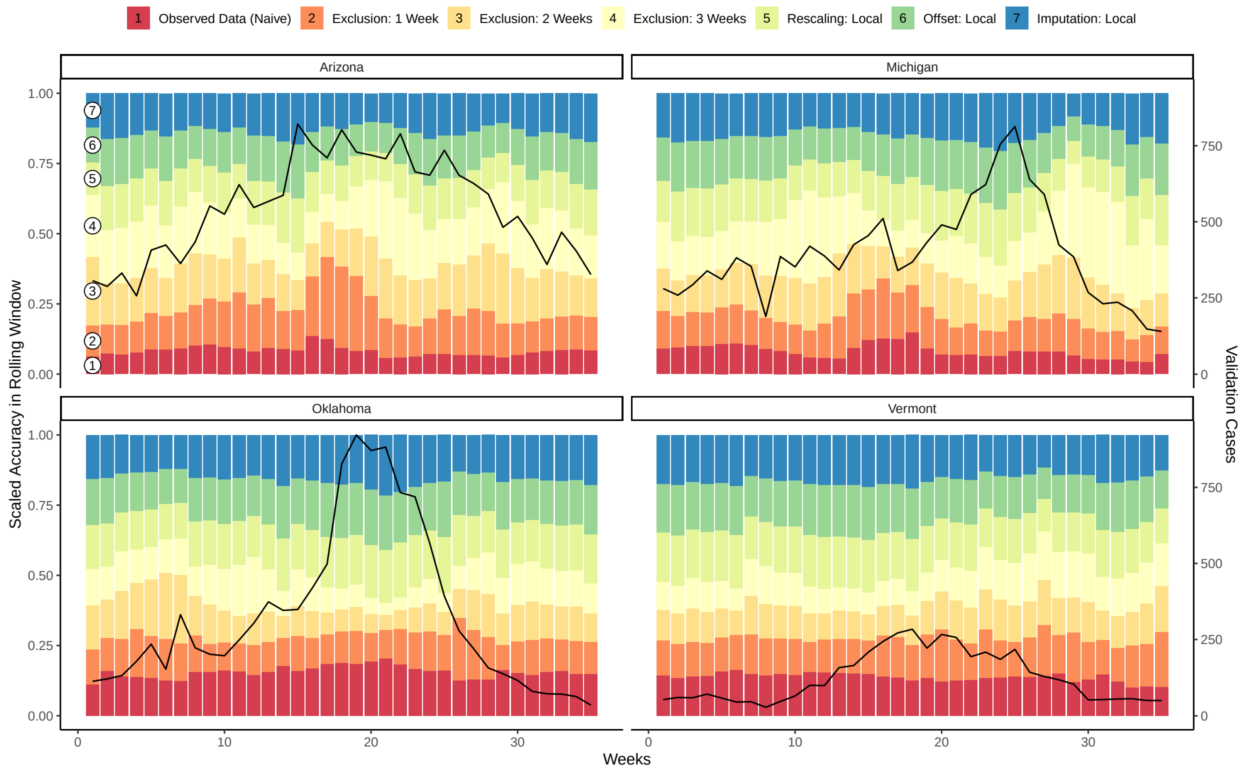

[1] Results based on absolute prediction error for 3 week rolling window centered at plotted week. Results for 35 weeks per state are shown.

Figure L: State-level ILI: Proportion of 50 weeks in which each of 7 methods performs best in terms of nowcasts and 1-week forecast weighted interval scores (ARMA model)

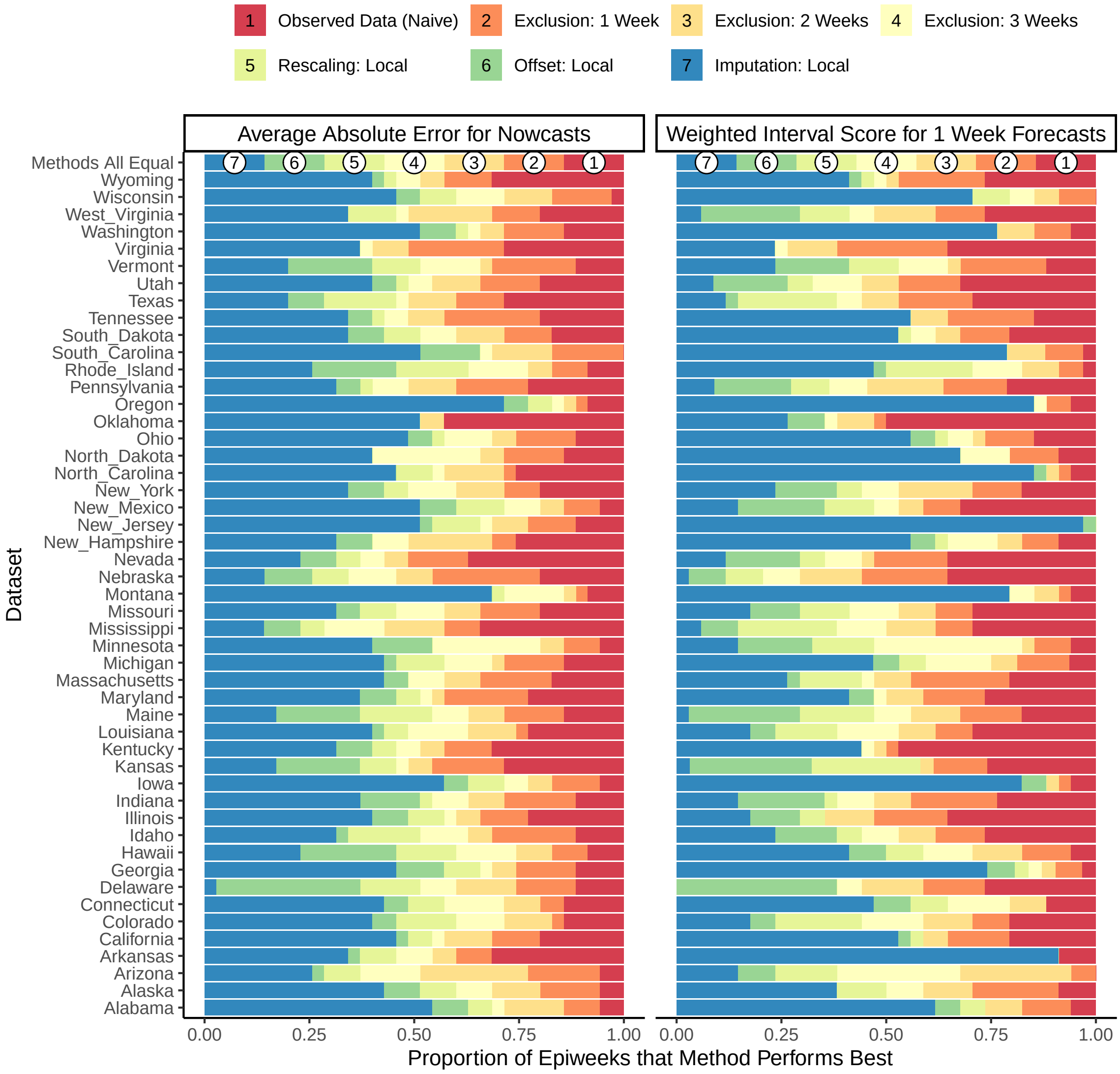

[1] Results for US national influenza are aggregated across 35 weeks in the 2018-2019 season. Florida is excluded from this analysis due to lack of data.

Figure M: Forecast bias and weighted interval scores for 3 weeks just before and just after season peak and for 3 weeks surrounding the season minimum/valley[1]

(a) Average bias in nowcasts and forecasts

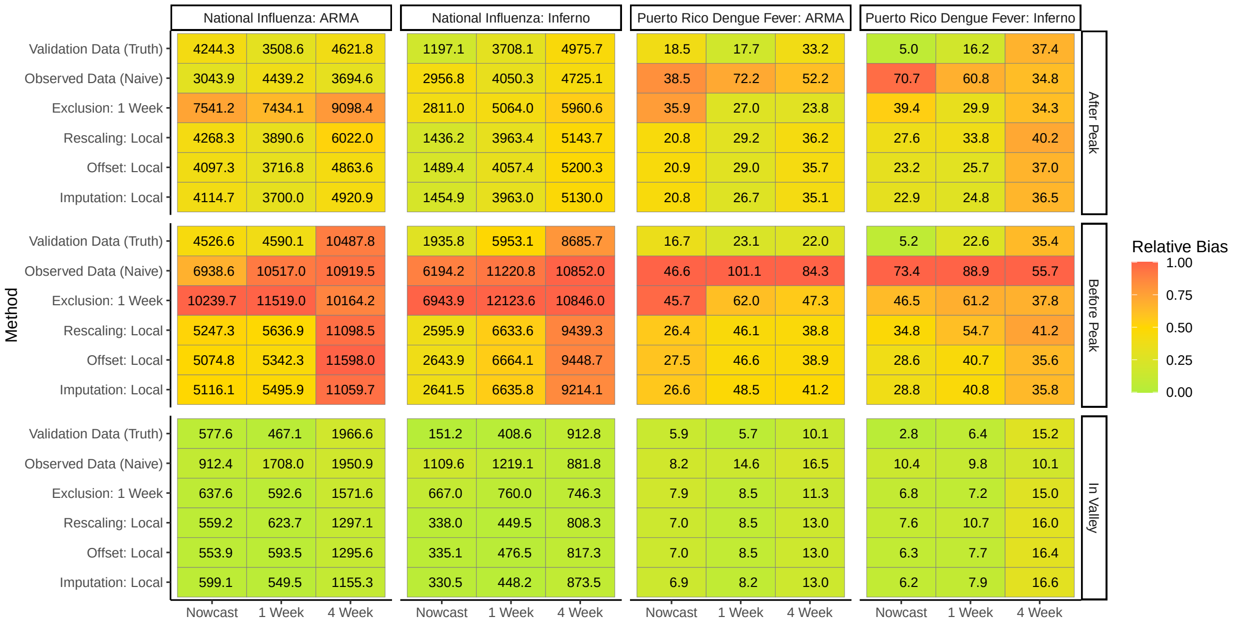

(b) Median weighted interval score (WIS) for forecasts

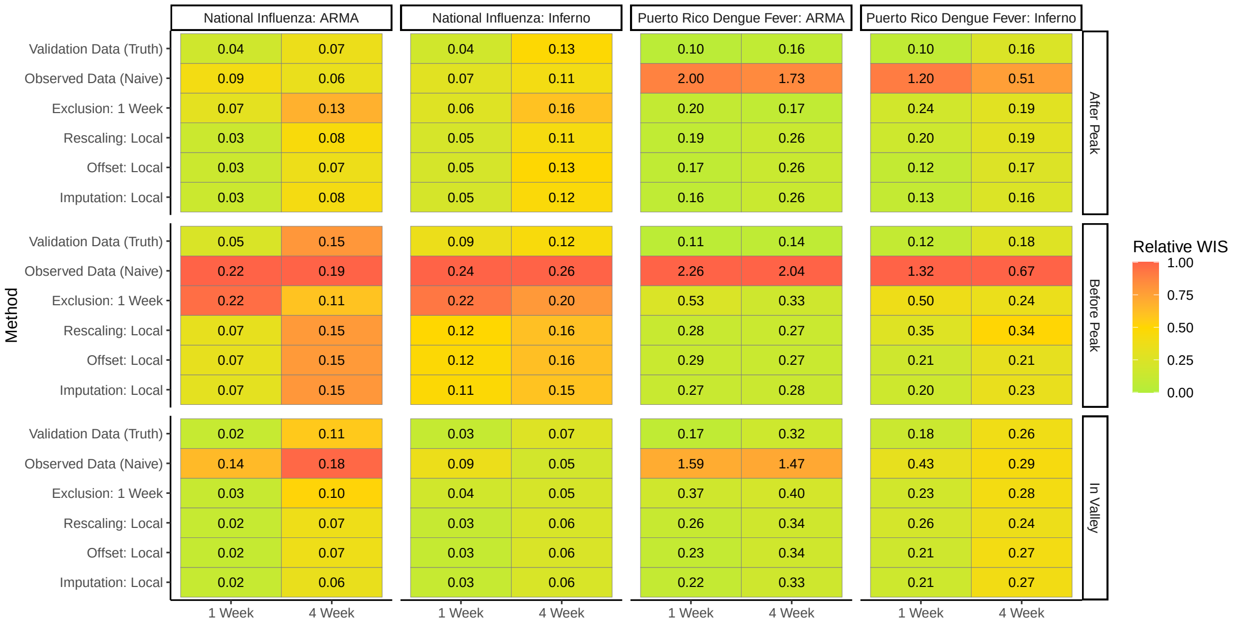

[1] Results based on aggregating nowcast and forecast performance for 3 weeks just before and 3 weeks just after each season's peak. Results also provided after aggregating 4 weeks before and after season minimum/valley. For ILI, the season valley corresponds to the minimum case counts within the first 35 weeks of the flu season.

6.3 Comparison of state ILI reporting factor estimators

In **Figure N**, we plot the estimated inverse reporting factor $\pi_{ts}(0)$ obtained for the lag-based and local estimation methods. We also plot the observed $N_{ts}(0)/N_{ts}(\infty)$ for each week. We note that the inverse reporting factor estimation methods aim to estimate the *expected* $\pi_{ts}(d) = E(\mathbf{N}_{ts}(d)/\mathbf{N}_{ts}(\infty))$ rather than the *observed* ratio based on data realizations $N_{ts}(d)$ and $N_{ts}(\infty)$. However, comparison of the estimated $\pi_{ts}(d)$ with the data realizations $N_{ts}(0)/N_{ts}(\infty)$ can provide insight into the accuracy of our estimates.

From this figure, it is clear that the lag-based method using past season reporting data substantially over-estimates $\pi_{ts}(0)$, while the local method resulted in much better estimations. This is due to a large discrepancy in the reporting practices in Vermont between the 2017-2018 and 2018-2019 seasons, described on average in **Figure C**. While less dramatic, we can see small improvements in estimated $\pi_{ts}(d)$ for other states as well. **Figure N** also demonstrates that the local estimation tends to produce noisier (i.e., more "wiggly") estimates of $\pi_{ts}(0)$ over t than the lag-based method. This is because the lag method uses data for two seasons of reporting, while the local method uses data from only the prior 15 weeks.

Figure N: Estimated $\pi_{ts}(0)$ obtained from different lag estimation methods for state-level ILI in the 2018-2019 flu season[1]

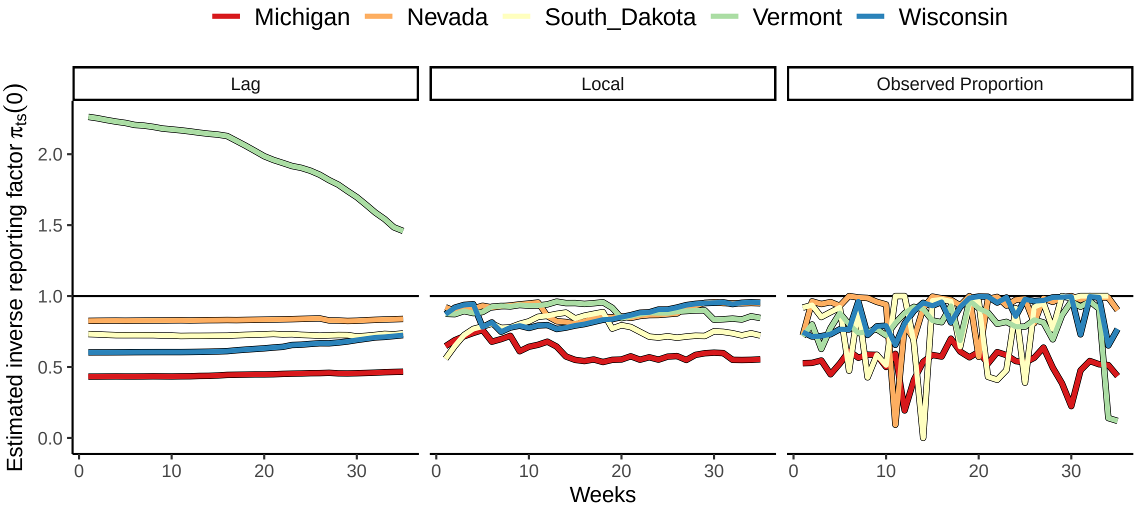

[1] Data were downloaded on June 13th, 2021. The local lag method used data from the previous 15 weeks to estimate the inverse reporting factors. The standard lag method used data from the previous 2 seasons to estimate the inverse reporting factors. *Observed* inverse reporting factors for each week are also plotted.

6.4 Comparative performance of observed and validation data analysis

indent **Figure P** takes a closer look at nowcast and forecast performance for ARMA and Inferno models applied to the observed real-time data. For nowcasting, the ARMA model tends to out-perform Inferno, but ARMA modeling performs very poorly for 1 week forecasting. This may be because the ARMA(2,2) model relies on the data from the last two weeks, which in this example are subject to reporting error. Since it takes at least two weeks for reporting to converge to validation values for these data, the most recent two weeks' real-time data will lead the ARMA modeling to expect a downward trajectory of case counts. This results in severe under-estimation in terms of forecasts. In contrast, Inferno uses data from the recent weeks but also borrows information from past seasons, which may help it better adapt to unexpected sharp decreases in real-time case counts due to reporting errors. This difference in forecast performance based on observed real-time data explains the method rankings results presented in the main paper, which show that observed data analysis has better comparative performance for Inferno modeling than for ARMA modeling, relative to the forecast performance of reporting correction methods.

Figure O: Relative accuracy (1/absolute prediction error, scaled across methods) of 1 week ahead forecasts for dengue fever using real-time or validation data for modeling[1]

(a) ARMA model

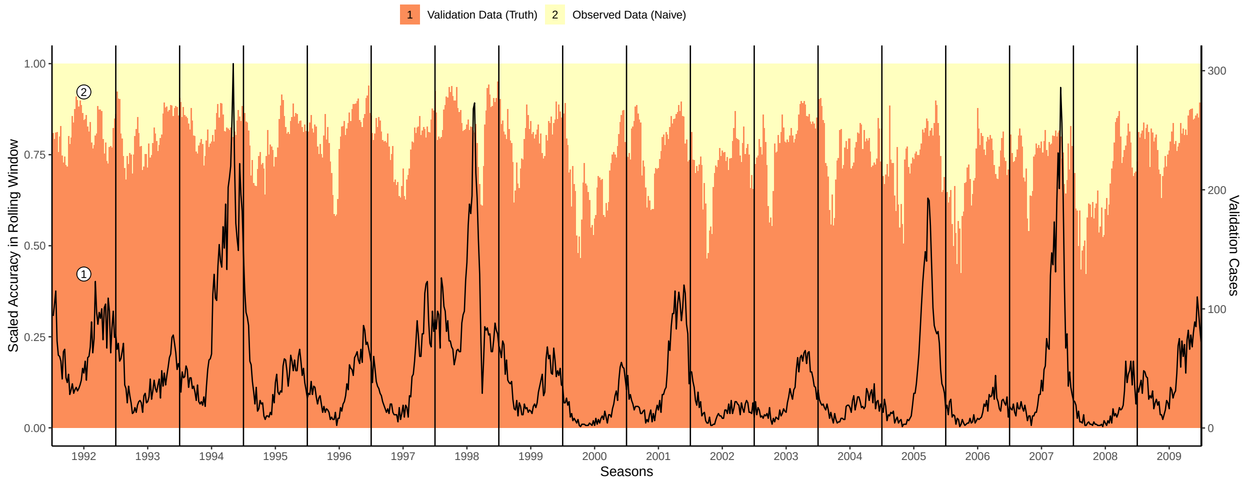

(b) Inferno model

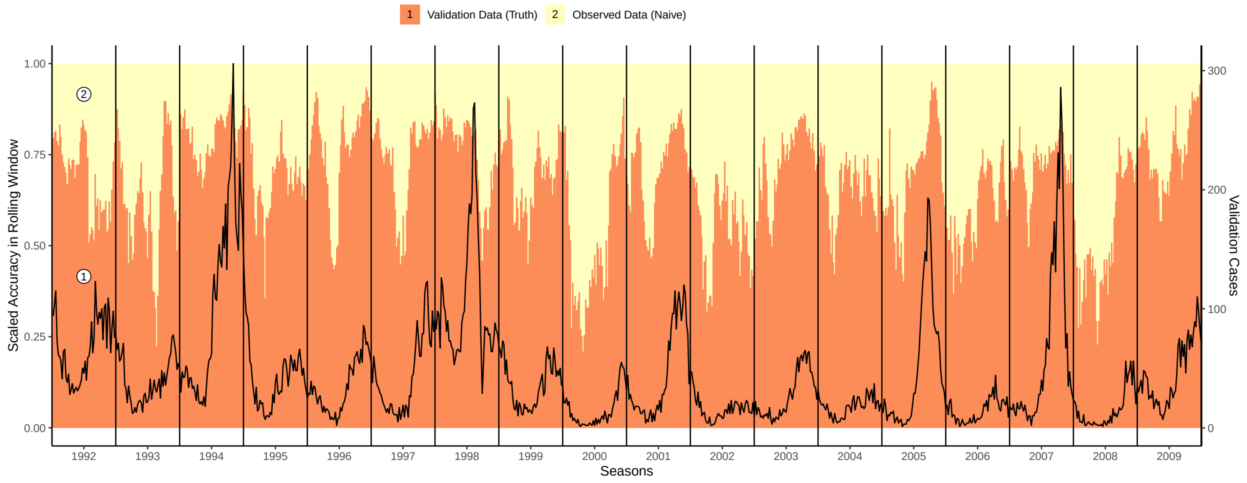

[1] Results based on absolute prediction error for 5 week rolling window centered at plotted week. Results for 50 weeks per season are shown. The black line represents observed validation case counts for each week.

Figure P: Dengue fever data nowcasts and forecasts from ARMA and Inferno modeling of observed data, compared to validation and initial case reports

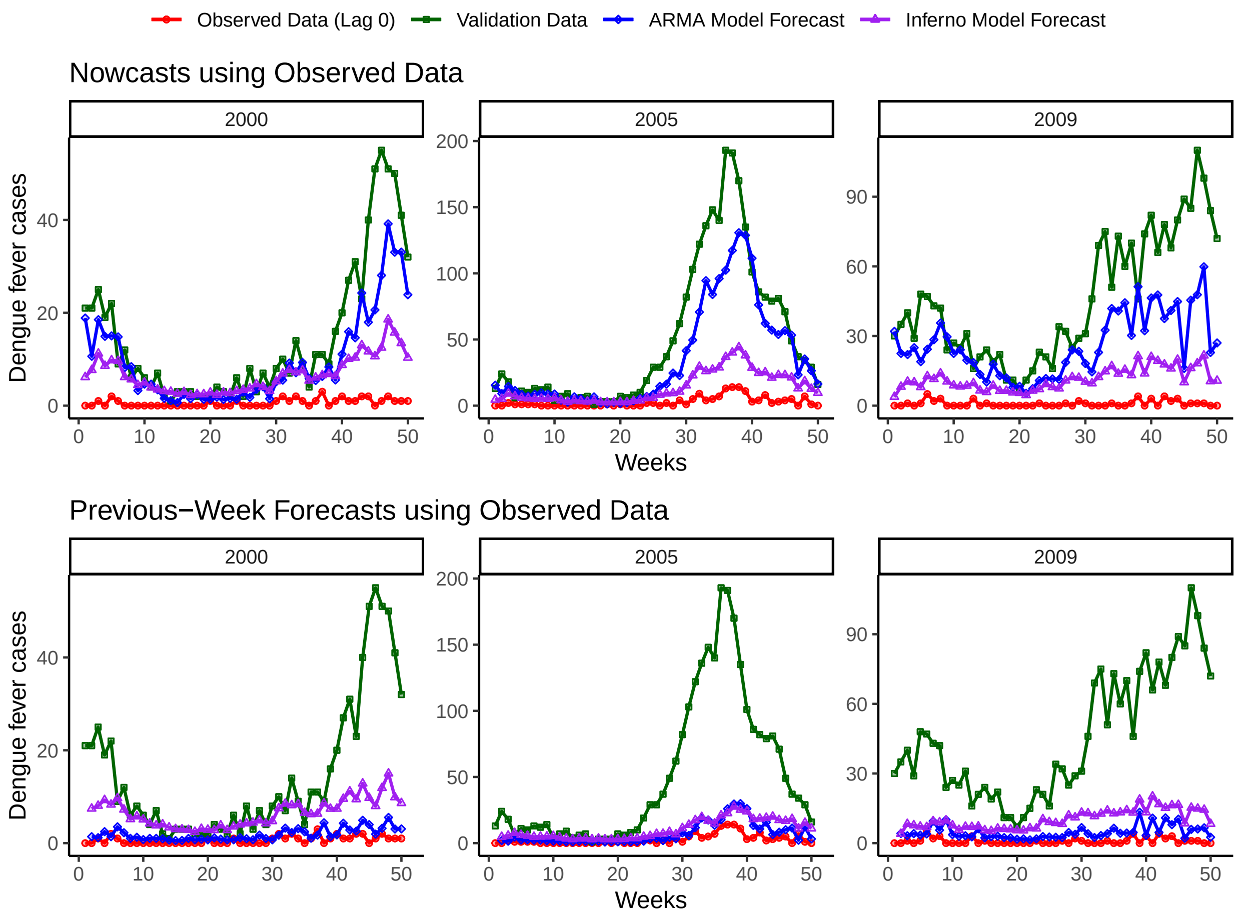

6.5 Impact of number of weeks included in local reporting estimation

In this section, we provide some additional figures exploring the impact of K on the performance of the local $\pi_{ts}(d)$ estimation method. **Figure Q** shows the $\pi_{ts}(0)$ estimates across K for national US ILI and Puerto Rico dengue fever. Corresponding nowcast and forecast errors are provided in **Figure R**. **Figure S** provides similar diagnostics for Vermont ILI, where reporting factor estimates and forecasts are obtained for the 2018-2019 flu season.

Figure Q: Impact of K on estimated $\pi_{ts}(0)$ using local estimation in dengue fever and national US ILI data (ARMA model)[1]

(a) Puerto Rico dengue fever

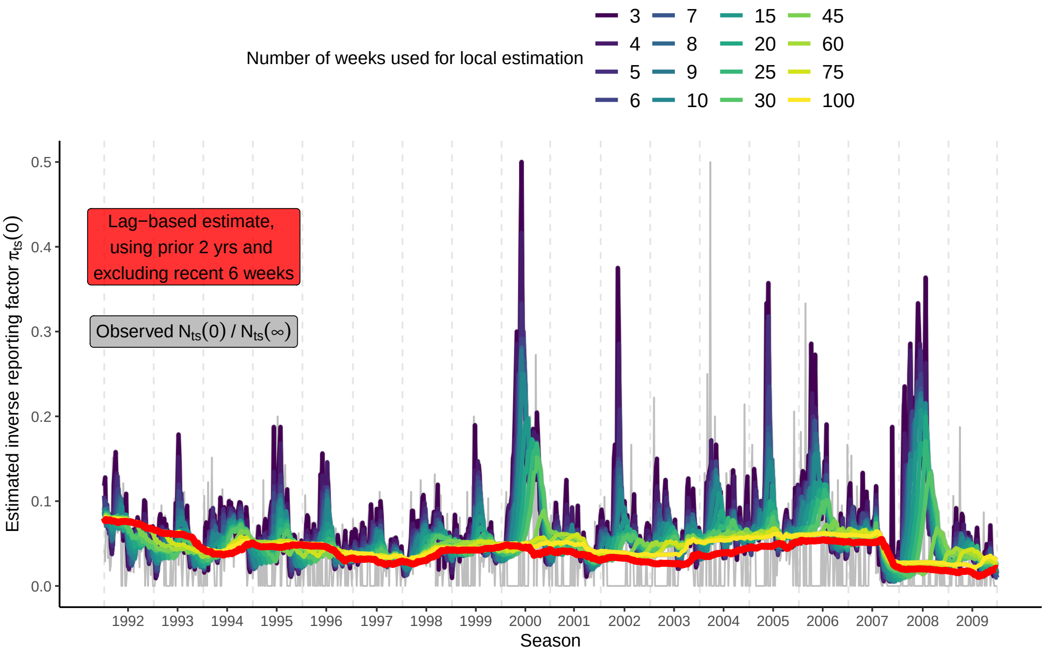

(b) National US Influenza-like Illness

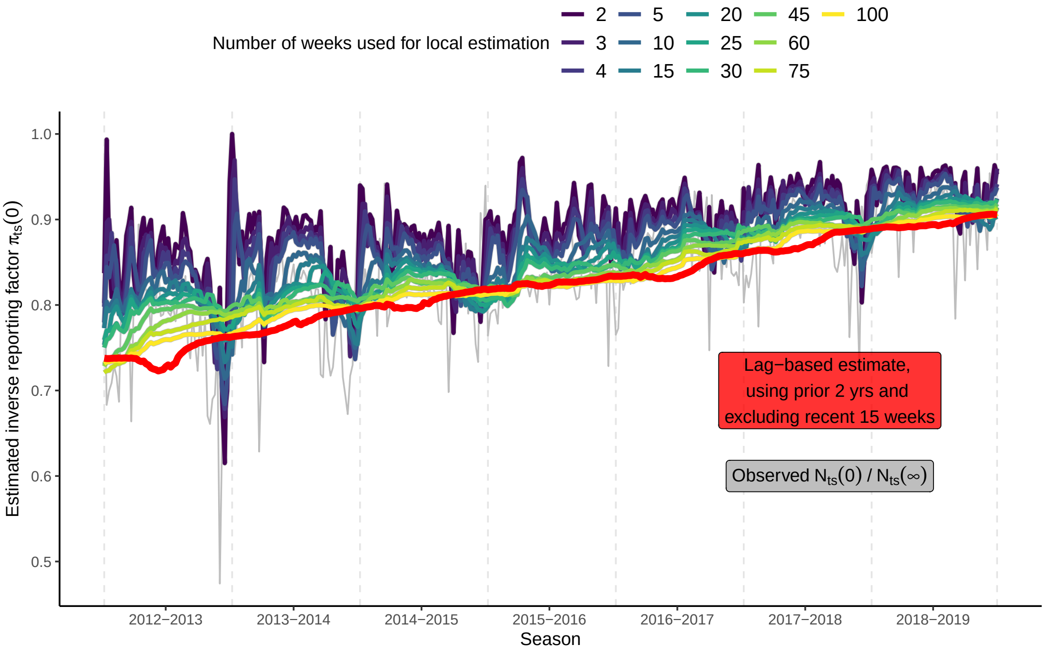

[1] Local estimates for $\pi_{ts}(d)$ are obtained using *Eq. 8*.

Figure R: Impact of local estimation K on nowcast/forecast errors in dengue fever and national US ILI data using rescaling method (ARMA model)[1]

(a) Puerto Rico dengue fever

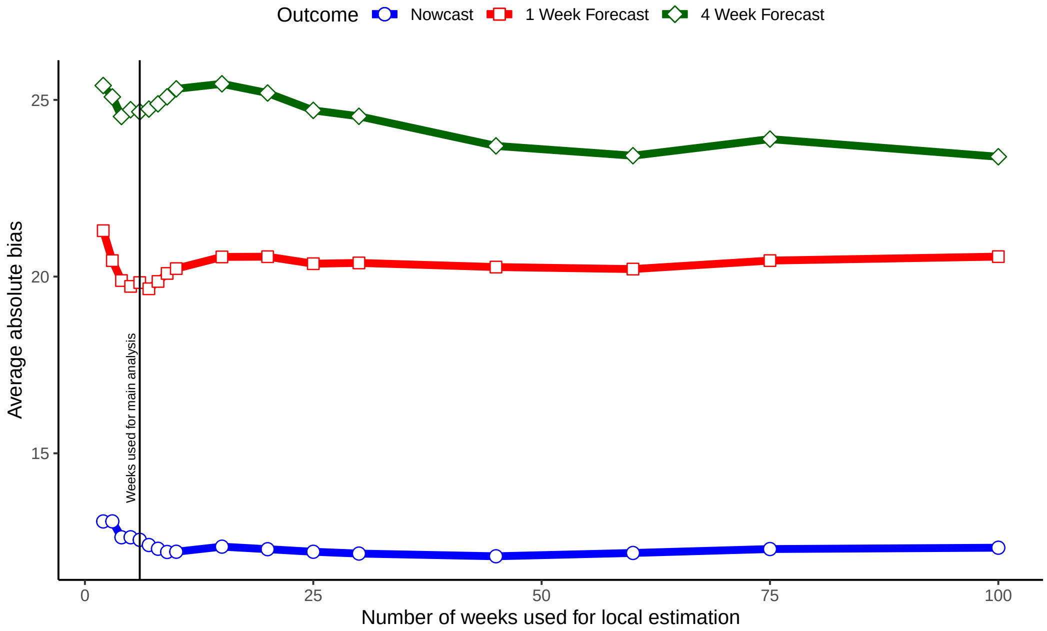

(b) National US Influenza-like Illness

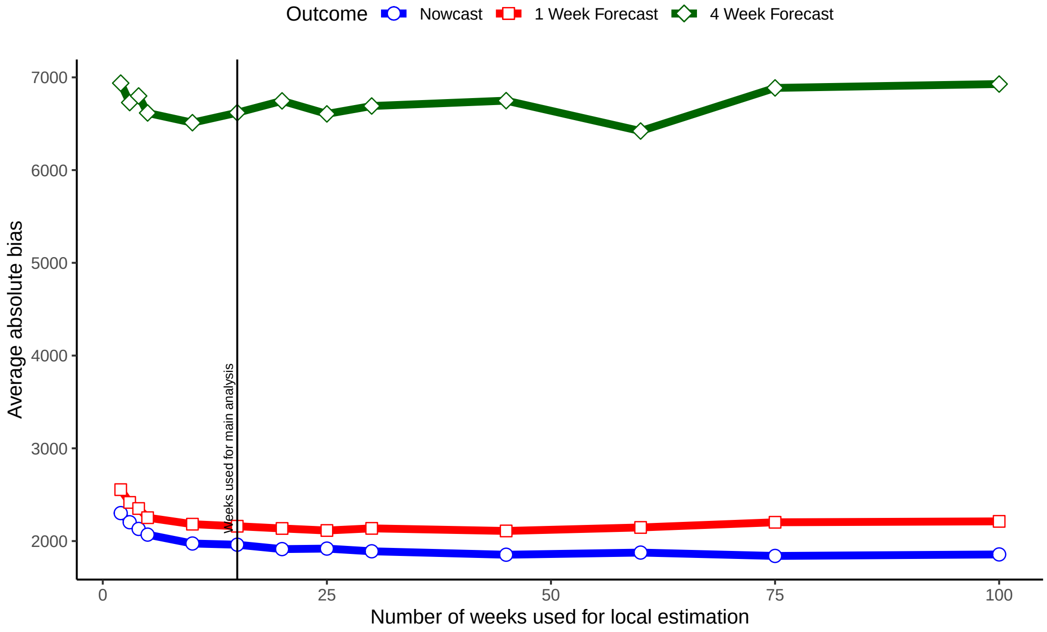

[1] Results based on aggregation across 50 weeks (dengue fever) or 35 weeks (influenza) across all seasons. Local estimates for $\pi_{ts}(d)$ are obtained using *Eq. 8*.

Figure S: Impact of K for local $\pi_{ts}(d)$ estimation for forecasting Vermont ILI in the 2018-2019 season (ARMA model)[1]

(a) Estimated $\pi_{ts}(0)$

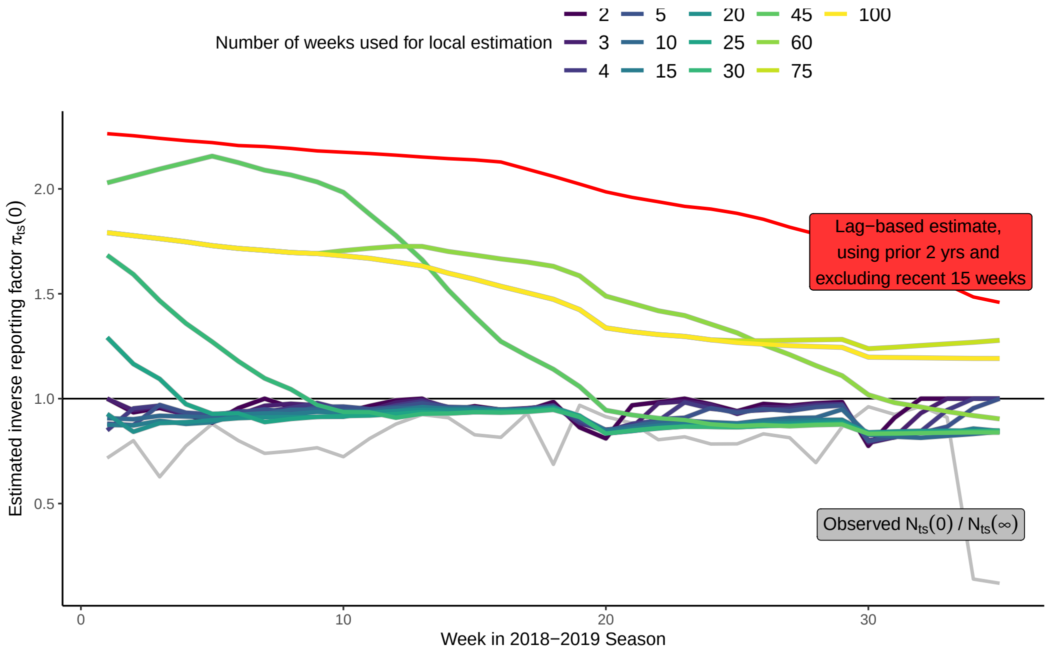

(b) Average absolute nowcast and forecast errors using rescaling method

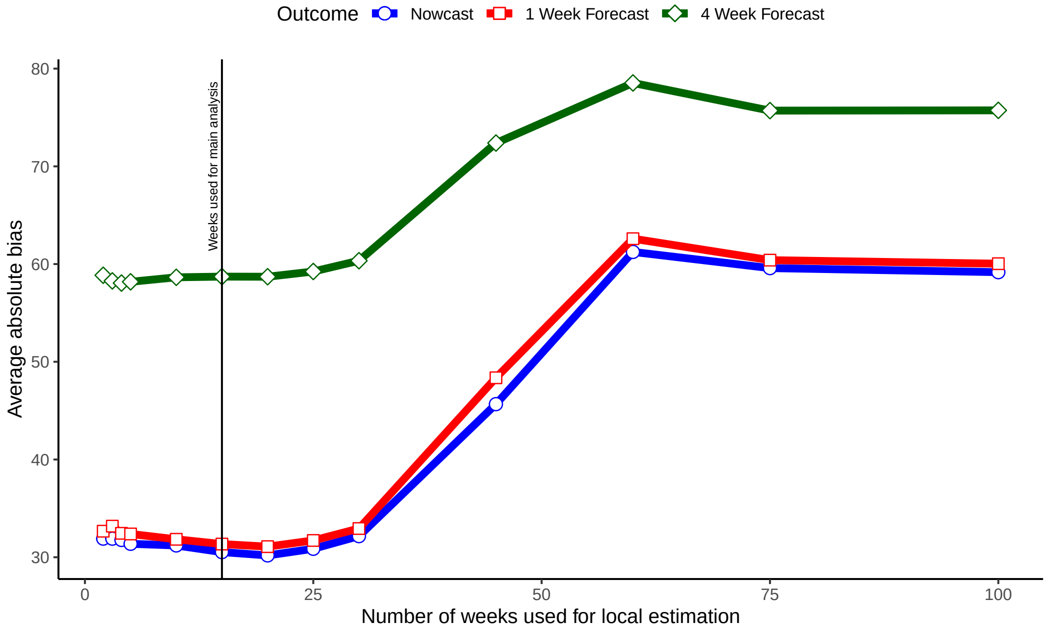

[1] Results based on aggregation across 35 weeks in the 2018-2019 season. Local estimates for $\pi_{ts}(d)$ are obtained using *Eq. 8*.

7 A note on lag scaling

Figure T presents simulation Scenario 5 average absolute nowcast and forecast prediction errors for (1) validation data analysis, (2) analysis of observed data, and (3) rescaling with correctly-specified reporting factors across different levels of reporting delay (a). Results are based on aggregates across 50 weeks of simulated 2009 data and 10 simulation datasets for each value of a (x-axis).

When validation data itself was used for estimation, the Inferno modeling tended to provide lower absolute nowcast error than ARMA modeling. ARMA and Inferno model provided more similar forecast errors based on validation data, with slightly better performance seen across a for Inferno. Inferno also out-performed ARMA across a in terms of forecasts, but this association flipped for nowcasts when uncorrected real-time data were used and when the amount of reporting delay was large (e.g. less than 80% of cases reported at lag 0). When we applied the lag-based rescaling method (using the correct reporting factors) to the observed data, ARMA results mimicked use of validation data in terms of nowcast performance. For Inferno, however, the rescaling method produced some residual bias in nowcasts, and this bias increased as the proportion of cases reported at lag 0 decreased. The rescaling method produced bias in forecasts for both Inferno and ARMA models when the amount of under-reporting was very large ($a < 0.3$).

To illustrate the source of this bias in the original dengue fever data, we compared nowcasts based on modeling the observed data wihout correction and after applying the rescaling and mean model offset methods (**Supp. Figure U**). For Inferno modeling, the rescaling method clearly showed instability, where nowcasts for individual weeks were occasionally very far from the validation values, far beyond what we saw using the uncorrected observed data. The mean model offset and imputation methods (not shown) produce much more stable nowcasts. When we probed this problem further, we discovered that this instability was being driven by small fluctuations in the observed data. In the setting with a low number of validation cases and a large rate of under-reporting, a single additional case in the observed data can translate into a very large impact on the rescaled data, which is input into the forecasting model. We would not expect this same instability if either the disease rate were higher or if the rate of under-reporting were smaller. It is the combination of these factors that produces the instability in the rescaling method shown here.

Figure T: Simulations: average absolute prediction error for nowcasts and 1 week forecasts by proportion of initially-reported cases in simulated 2009 dengue fever data [1]

(a) Nowcasts

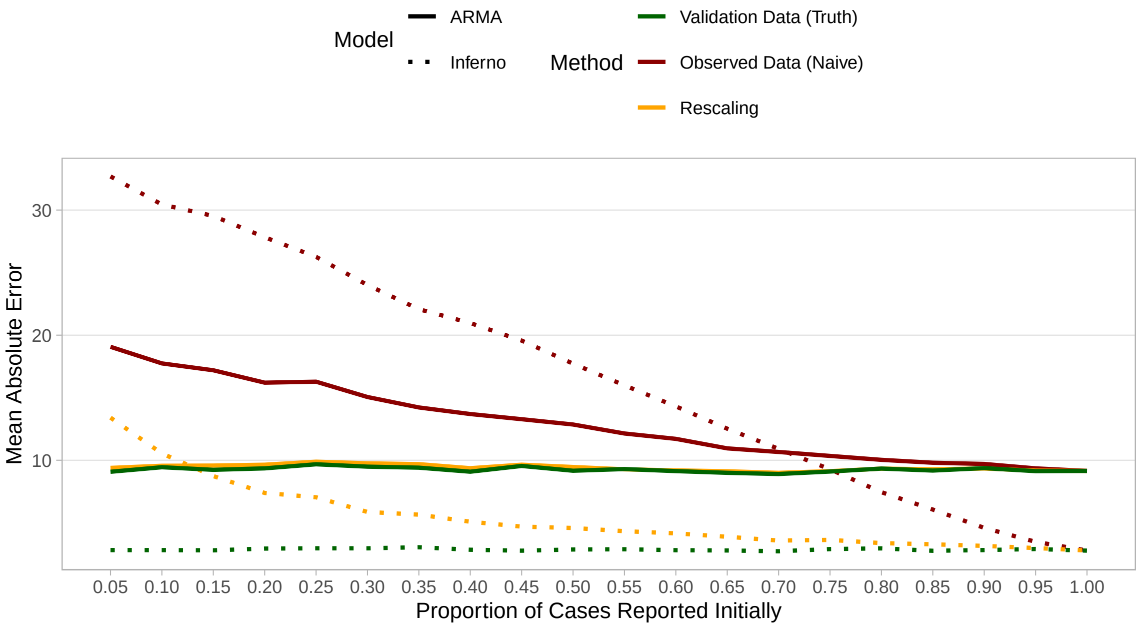

(b) Forecasts

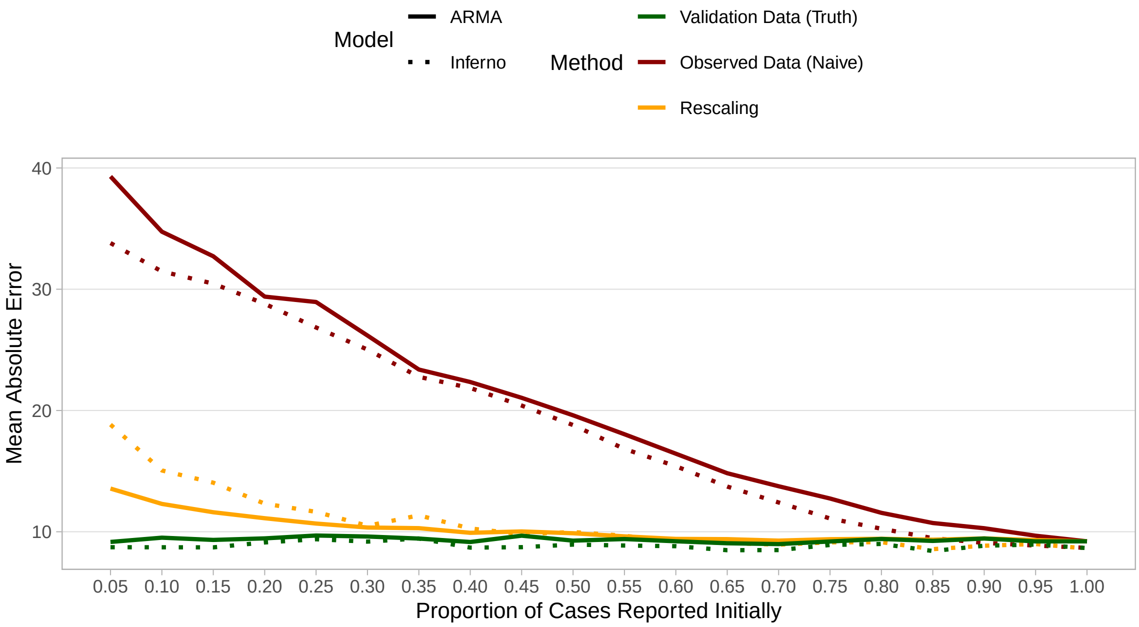

[1] Results aggregated across 50 weeks and 10 simulation replicates for each proportion.

Figure U: Data Analysis: nowcast prediction error in actual dengue fever data for 2009

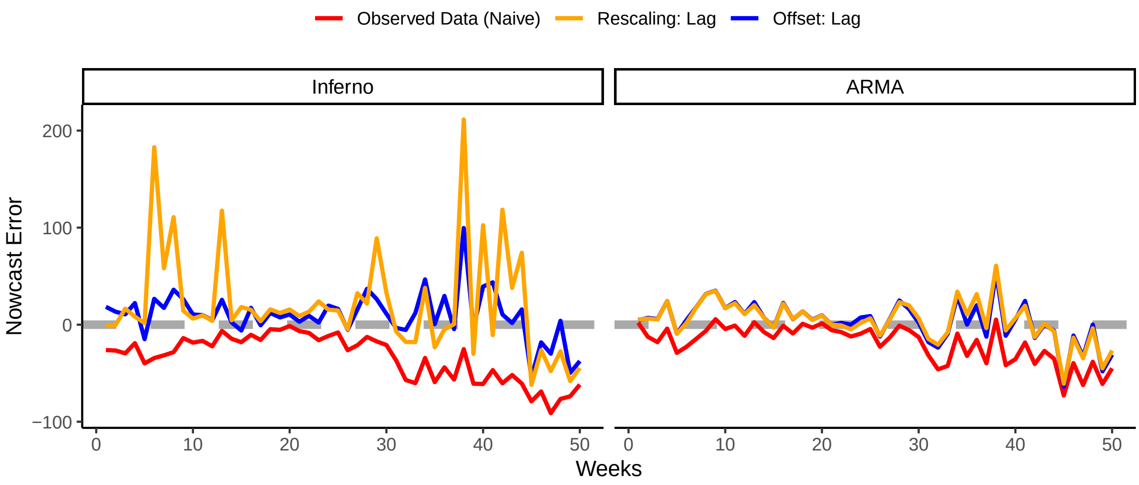

[1] All values represent differences between nowcast and true validation values. The gray line corresponds to equality with validation data.

8 Simulations of dengue fever data

Figure Va provides a visualization of the 200 simulated validation dengue fever datasets used in the simulation study. The mean structure and variability of these simulated datasets were chosen to mimic the actual data on dengue fever cases in the Puerto Rico between 1990 and 2009, shown in the figure by a thicker black line.

As described in the main paper, reported delay was then generated under various simulation scenarios. For simplicity, all reporting profiles considered followed the following form: $\pi_{ts}(d = \{0, ..., 6\}) = \{a, 0.5 + a/2, 0.75 + a/4, 13/16 + 3a/16, 14/16 + a/8, 15/16 + a/16, 1\}$. This structure was parameterized in terms of constant a, the proportion of eventually-reported cases that were reported initially at lag week 0. Corresponding $\pi_{ts}(d)$ are plotted for a in (0.05, 1) in **Figure Vb**.

Results are presented in the main paper, and we present some additional results here. **Figure W** provides the estimated weighted interval scores for the 2009 simulated data. **Figure Y** explores the performance of the proxy shrinkage strategy for estimating $\pi_{ts}(d)$ and applying the rescaling method in terms of resulting forecast performance as a function of (1) the quality of the proxy in terms of its correlation to $N_{ts}(\infty)$ and (2) the amount of under-reporting (i.e., a). **Figure X** explores forecast performance for the exclusion method as a function of the amount of under-reporting.

Figure V: Visualization of simulated dengue fever data

(a) Validation data for 200 simulated datasets and observed dengue fever data (black line)

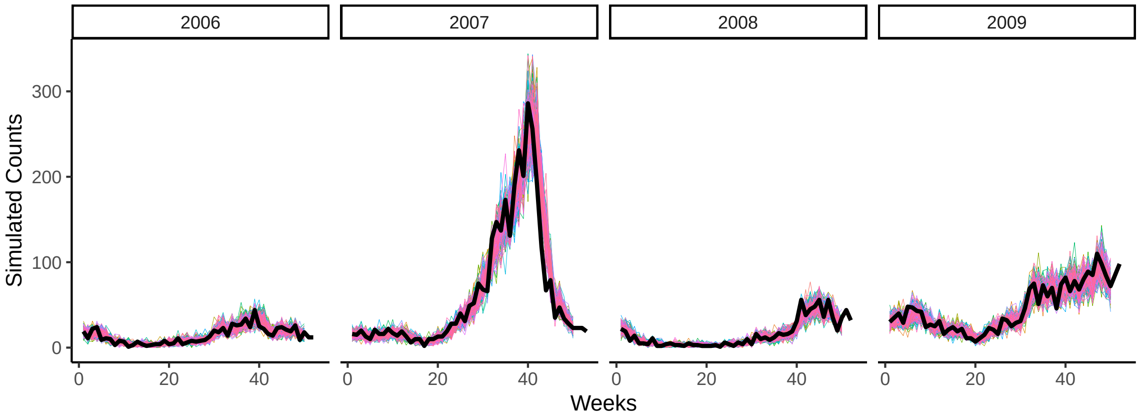

(b) Inverse reporting factors as a function of a and lag

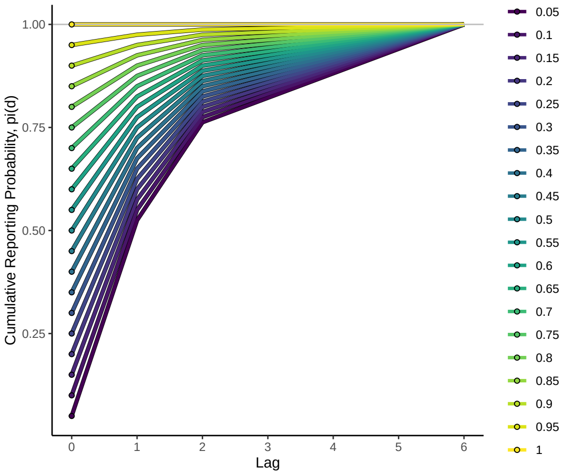

Figure W: Median forecast weighted interval scores in dengue fever simulated data (ARMA models, Scenarios 1-4) [1]

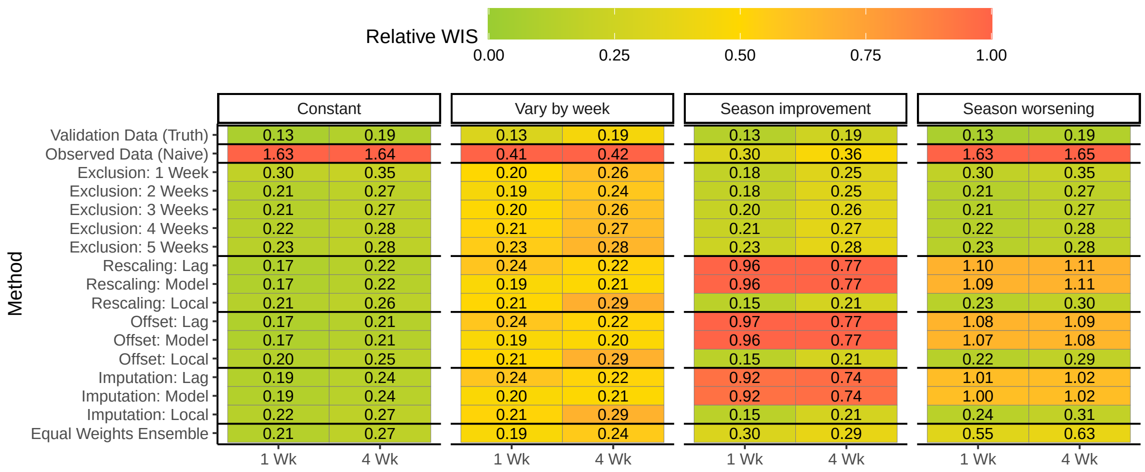

[1] Results correspond to the 2009 simulated data and are aggregated across 100 simulated datasets and 50 weeks. Relative weighted interval scores (WIS) are calculated relative to the largest value in each column.

Figure X: Forecast performance of exclusion method by proportion of initially-reported cases in dengue dever simulated data (ARMA models, Scenario 5)[1]

(a) Average absolute error for 1 week forecasts, scaled by season peak case counts

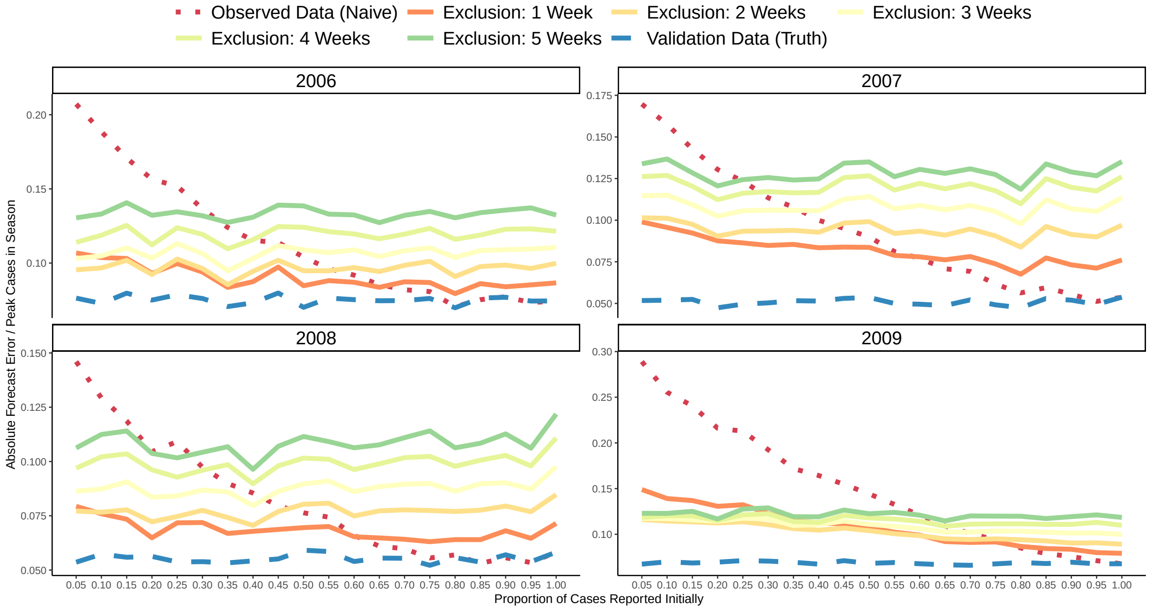

(b) Weighted interval score for 1 week forecasts

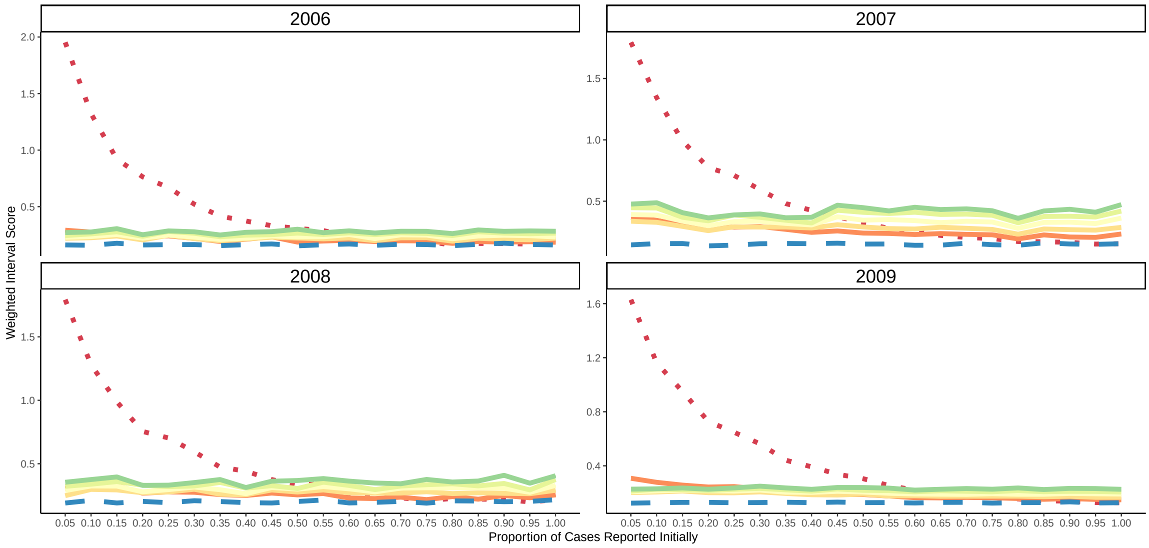

[1] Results aggregated across 50 weeks and 10 simulation replicates for each season/proportion.

Figure Y: Forecast performance of rescaling method with reporting factors based on proxy shrinkage by proxy quality and proportion of initially-reported cases in dengue fever simulated data (ARMA models, Scenario 5)[1]

(a) Average absolute error for 1 week forecasts, scaled by season peak case counts

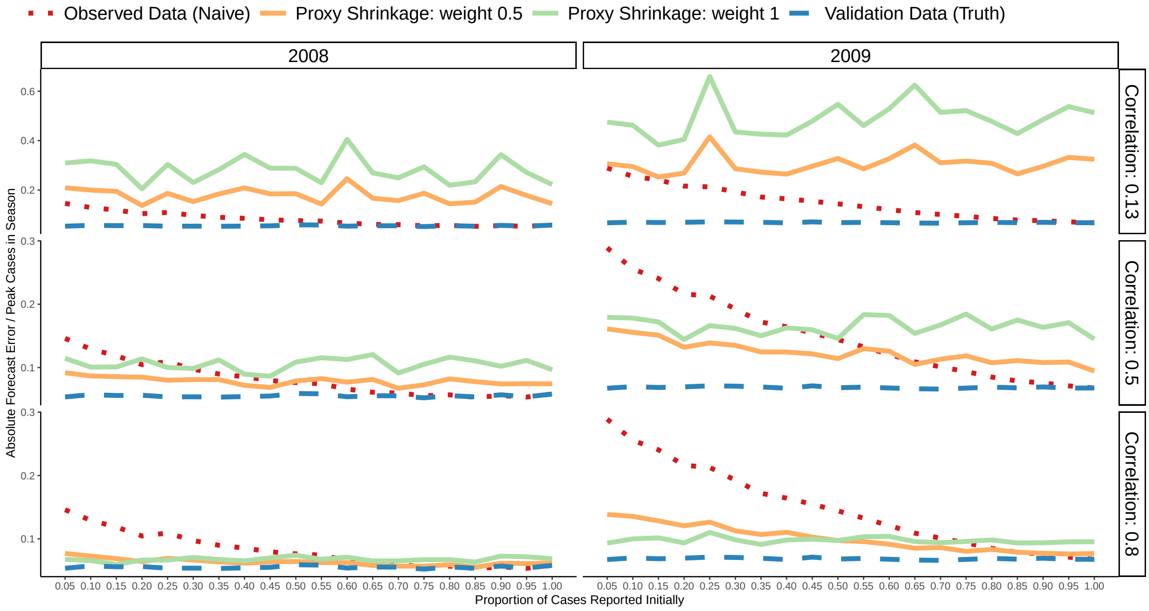

(b) Weighted interval score for 1 week forecasts

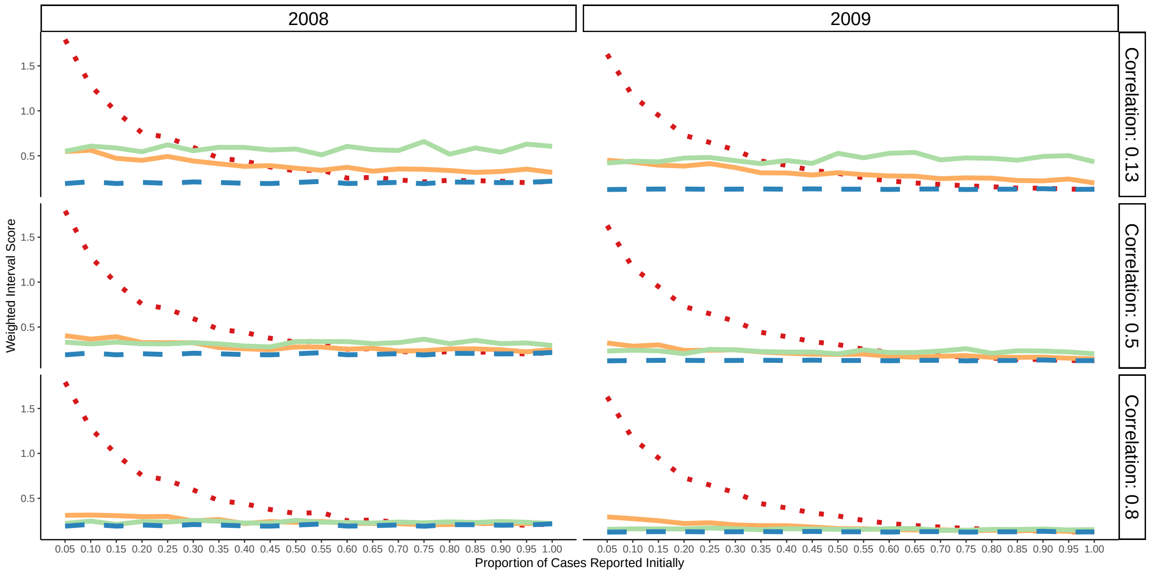

[1] Results aggregated across 50 weeks and 10 simulation replicates for each season/proportion.

9 Simulations of national US ILI data

In this section, we replicate the simulations presented for dengue fever-like data in the main paper and in **Section 8**. This time, however, we simulate data to look like the US national ILI dataset. These data are simulated exactly as described for the dengue fever setting in the main paper except reporting delay was simulated to be much less severe. We chose to limit our focus to the setting with higher initial reporting rates to better align with observed rates of reporting for these data. Recall, a denotes the proportion of eventually-reported cases that are reported initially (lag 0).

We considered the following simulation scenarios:

1. *Constant*: reporting was constant in t and s and corresponded to $a = 0.80$.

2. *Vary by week*: reporting varied by t and was constant in s. a initially increased from 0.80 to 1 during weeks 1 to 25 in each season and then decreased from 1 to 0.80 thereafter.

3. *Moderate improvement between seasons*: reporting improved in the last season, with $a = 0.80$ for the 2010-2017 flu seasons and $a = 1$ for the 2018 flu season.

4. *Moderate worsening between seasons*: reporting worsened in the last season, with $a = 1$ for the 2010-2017 flu seasons and $a = 0.80$ for the 2018 flu season.

5. *All season combinations*: Each strata of 10 simulation replicates was assigned a different value for a between 0.80 and 1. Within each strata, reporting (i.e., a) was constant in s and t.

For each set of simulated validation data, we also simulated 4 external proxy variables such that $p_{ts} = 2\log(N_{ts}(\infty) + 0.1) + e_{ts}$, where $e_{ts} \sim N(0, \sigma^2)$ and where σ^2 took values in (0.01, 1, 4, 16). These error rates corresponded to correlations between transformed p_{ts} and $N_{ts}(\infty)$ of 0.99, 0.80, 0.50, and 0.13. Results are aggregated across the first 35 weeks of the flu season.

Aggregated nowcast and forecast performance in terms of bias, WIS, and coverage of 95% confidence intervals are shown in **Figures Z and AA**. We find that the negative impact of changes in reporting delay between seasons is much less striking than for the simulated dengue fever results. This is largely due to the magnitude of the assumed differences in reporting between seasons, which in the current simulations are assumed to differ from 80% reporting on the first week to 100% reporting on the first week. Even when true reporting varied within each season by week, the local estimation method produced the best forecast performance, surpassing the model-based method where intra-season trends were modeled via a regression model. This is likely due to misspecification of the dependence on week t in the reporting delay model for the model-based approach. In general, the local estimation method produced the most robust performance across the different simulation settings. Interestingly, the imputation strategy often produced comparable or slightly worse performance than the rescaling and offset methods. We believe this is due to the extra forecast uncertainty resulting from the imputation procedure, which outweighs bias due to misspecified reporting factors in the setting where reporting is fairly good. This contrasts results seen in the dengue fever simulations, where the imputation approach resulted in improved nowcast and forecast coverage.

Figure BB compares the performance of the exclusion method for generating 1 week forecasts as a function of the number of excluded weeks K and the proportion of eventually-reported cases reported at lag 0. For all K and $\pi(0)$ greater than roughly 0.85, we find that excluding recently-reported weeks reduces forecast performance compared to ignoring reporting delay entirely. Unlike the dengue fever example, however, we do start to see some benefit of excluding one or two recent weeks for $\pi(0)$ less than 0.85. This may be because the higher case counts in the ILI setting makes the forecasting more reliable several weeks in the future, and the additional noise introduced by excluding recent weeks' data is less than the noise resulting from

the reporting delay.

Figure CC compares the performance of the proxy shrinkage for generating 1 week forecasts as a function of the proxy correlation with validation case counts and the proportion of eventually-reported cases reported at lag 0. Unless the correlation between the proxy and the validation case counts is very strong, we find that the proxy-based estimates of $\pi_{ts}(d)$ introduce more noise into the forecasting than the reporting delay itself does for reporting rates at least 80% in the first week. When initial case reporting gets worse, however, we expect to see greater advantage to including the proxy data.

Figure Z: Average nowcast and forecast biases and coverage of 95% confidence intervals for US ILI simulated data (ARMA models, Scenarios 1-4) [1]

(a) Average bias in nowcasts and forecasts

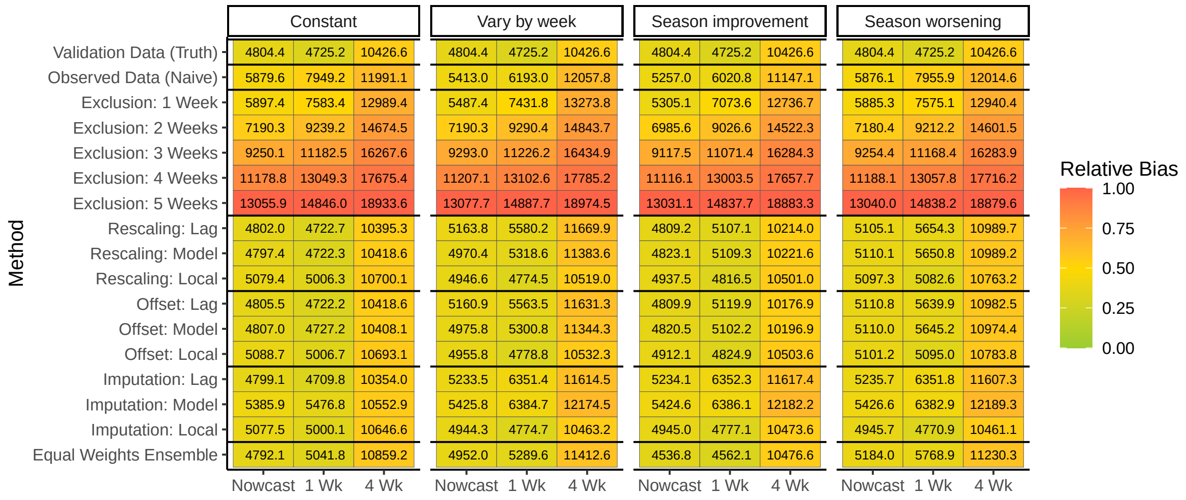

(b) Coverage of 95% confidence intervals

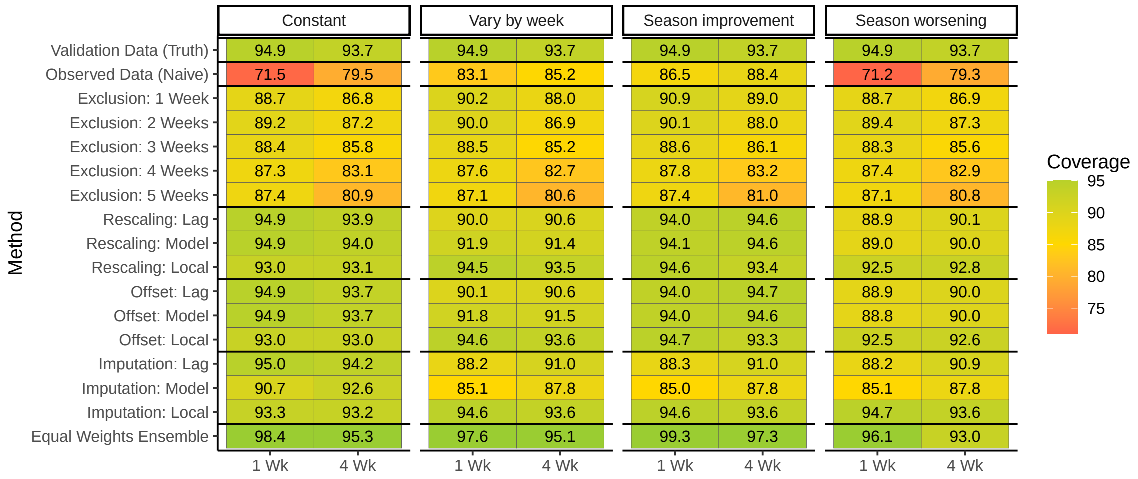

[1] Results correspond to the 2018 flu season simulated data and are aggregated across 100 simulated datasets and 35 weeks. Relative biases are calculated relative to the largest value in each column.

Figure AA: Median forecast weighted interval score for US ILI simulated data (ARMA models, Scenarios 1-4) [1]

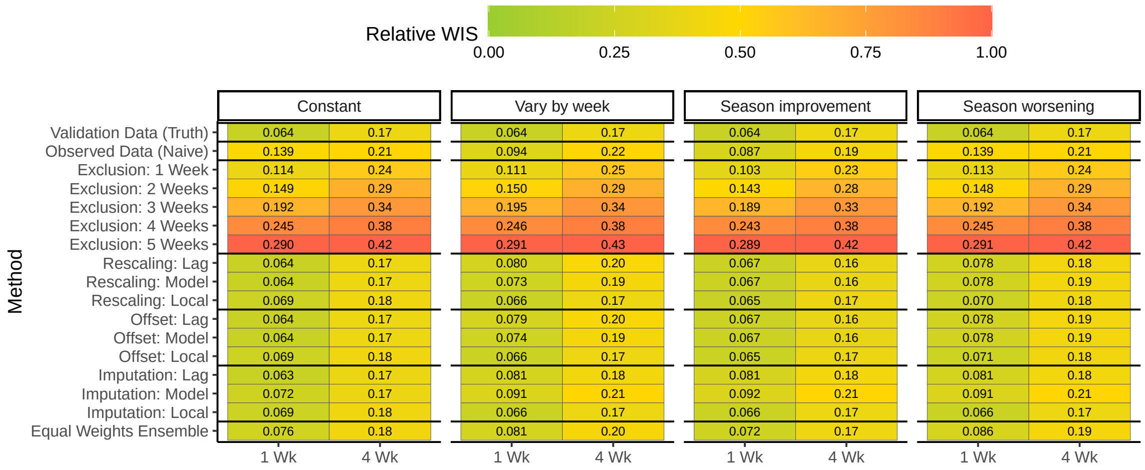

[1] Results correspond to the 2018 flu season simulated data and are aggregated across 100 simulated datasets and 35 weeks. Relative weighted interval scores (WIS) are calculated relative to the largest value in each column.

Figure BB: Forecast performance of exclusion method by proportion of initially-reported cases in ILI simulated data (ARMA models, Scenario 5)[1]

(a) Average absolute error for 1 week forecasts, scaled by season peak case counts

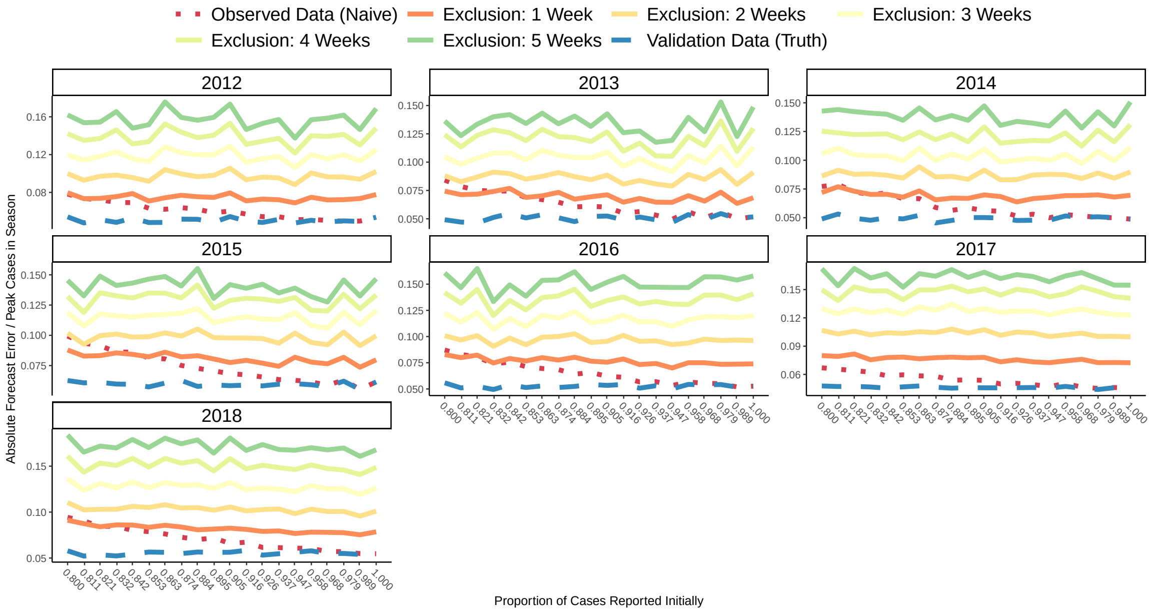

(b) Weighted interval score for 1 week forecasts

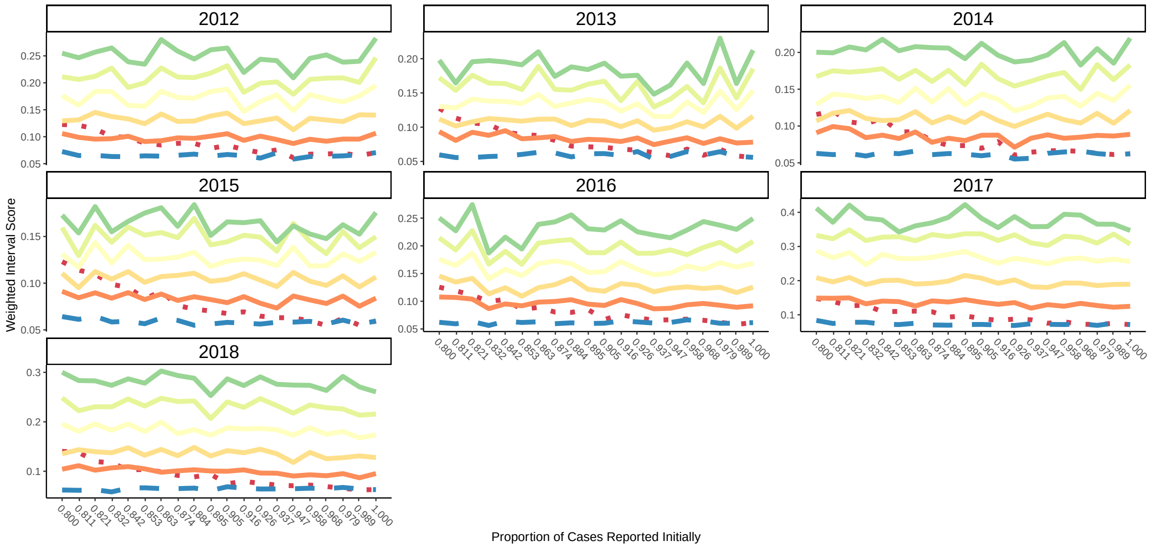

[1] Results aggregated across 35 weeks and 10 simulation replicates for each season/proportion.

Figure CC: Forecast performance of rescaling method with reporting factors based on proxy shrinkage by proxy quality and proportion of initially-reported cases in ILI simulated data (ARMA models, Scenario 5)[1]

(a) Average absolute error for 1 week forecasts, scaled by season peak case counts

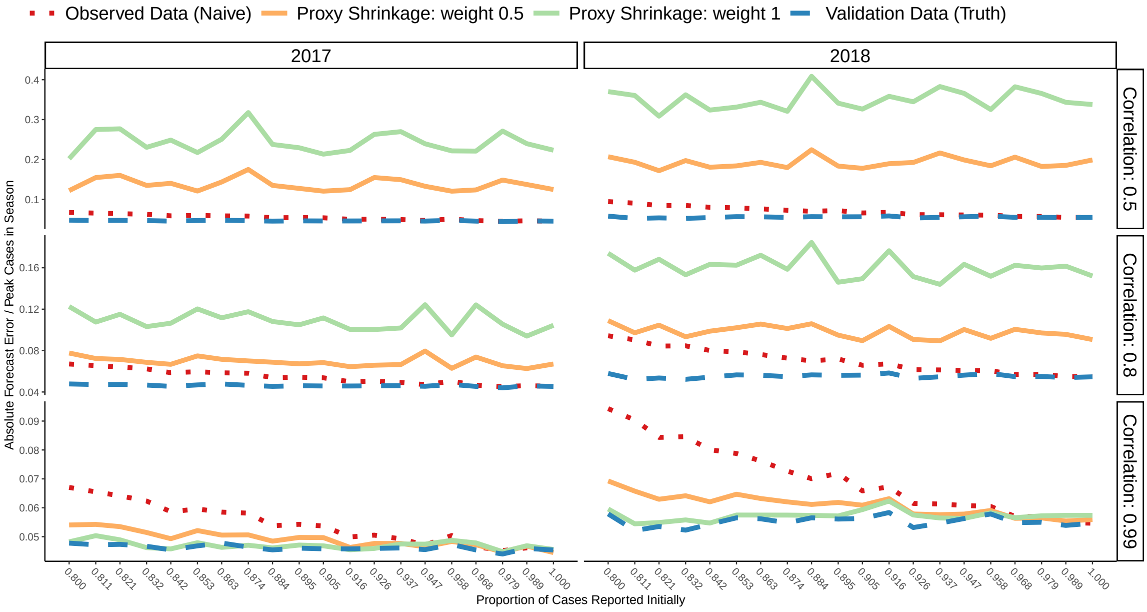

(b) Weighted interval score for 1 week forecasts

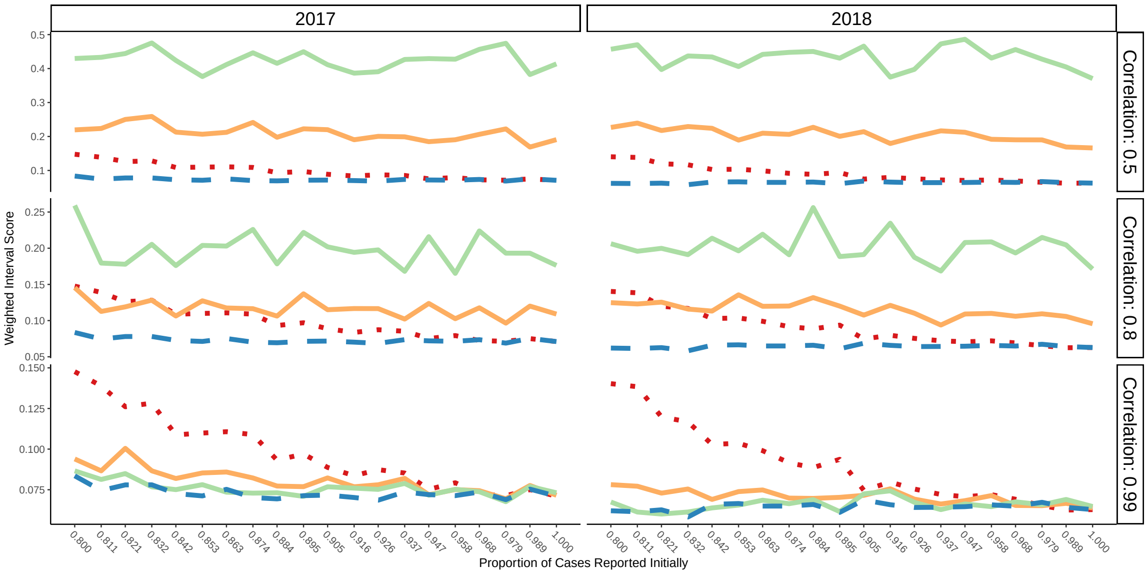

[1] Results aggregated across 35 weeks and 10 simulation replicates for each season/proportion.

10 Extension for modeling percent ILI among outpatient visits

The proportion of disease cases in some defined group of people is a common forecasting target. For example, the proportion of outpatients with influenza-like symptoms is a key metric used in monitoring ILI within the US. When the number of people seeking outpatient treatment (i.e., the denominator of the proportion) is not a fixed value, the leap between forecasting total cases and proportions of cases is not straightforward. In this section, we provide some intuition for how we could approach reporting delay correction when the target estimand for forecasting is a percent rather than the total number of cases. Many of the conceptual approaches in **Figure 2** in the main paper still apply, after modification. For illustration, we will focus on the setting where the goal is forecasting proportion ILI among outpatients in the US, but these methods can be applied more generally.

Methods

Let $y_{ts}(d)$ be the proportion of outpatients with ILI symptoms for week t in season s at lag d, and let $y_{ts}(\infty)$ denote the corresponding validation proportion. $y_{ts}(d)$ is similar to our previously-defined $N_{ts}(d)$, except it has been divided by the total number of outpatients reported for week t and season s by lag d. We can reframe the problem of addressing reporting delay in terms of selection bias, where the case rate in the *sample* of outpatients reported by week d may or may not be representative of the eventual sample of outpatients included in the validation calculation. We expect the intermediate sample reported by lag d to differ from the validation sample in two ways (1) some patients in the intermediate sample may be excluded from the validation sample and (2) some patients in the validation sample may not be included in the intermediate sample. While (2) is driven by reporting delay, we observe that (1) can also occur in the US national ILI data, so we want our methods to be flexible enough to handle both situations.

Consider the broader (possibly poorly-defined) population that is eligible to become an outpatient included in our analysis, due to geographic area of residence or otherwise. For each *individual* in that population, we define an indicator for whether or not they will be included in the validation sample as an outpatient, $S_{ts}(\infty)$. We also define an indicator for their hypothetical ILI symptom status, D_{ts}. We define similar quantities $S_{ts}(d)$ indicating whether or not each individual was included in the intermediate sample used to estimate $y_{ts}(d)$. We can then rewrite $y_{ts}(\infty) = P(D_{ts} = 1|S_{ts}(\infty) = 1)$ and $y_{ts}(d) = P(D_{ts} = 1|S_{ts}(d) = 1)$. We can relate these two quantities as follows:

$$
\begin{aligned}
y_{ts}(d) &= \frac{P(S_{ts}(d) = 1|D_{ts} = 1)P(D_{ts} = 1)}{P(S_{ts}(d) = 1|D_{ts} = 1)P(D_{ts} = 1) + P(S_{ts}(d) = 1|D_{ts} = 0)P(D_{ts} = 0)} \qquad (Eq\ i) \\
&= \frac{r_{ts}(d)P(D_{ts} = 1)}{r_{ts}(d)P(D_{ts} = 1) + P(D_{ts} = 0)} \\
&= \frac{p_{ts}(d)y_{ts}(\infty)}{p_{ts}(d)y_{ts}(\infty) + \{1 - y_{ts}(\infty)\}}
\end{aligned}
$$

where we define

$$
\begin{aligned}
r_{ts}(d) &= \frac{P(S_{ts}(d) = 1|D_{ts} = 1)}{P(S_{ts}(d) = 1|D_{ts} = 0)} \qquad (Eq\ j) \\
r_{ts}(\infty) &= \frac{P(S_{ts}(\infty) = 1|D_{ts} = 1)}{P(S_{ts}(\infty) = 1|D_{ts} = 0)} \\
p_{ts}(d) &= \frac{r_{ts}(d)}{r_{ts}(\infty)}
\end{aligned}
$$

Inverting *Eq i*, we have that

$$y_{ts}(\infty) = \frac{y_{ts}(d)}{p_{ts}(d) + y_{ts}(d)\,[1 - p_{ts}(d)]} \qquad (Eq\ k)$$

In the following text, we describe a two-step estimation process for estimating $p_{ts}(d)$ and forecasting future values of $y_{ts}(\infty)$. Similar to methods described in the main paper, we can estimate $p_{ts}(d)$ using historical data on real-time proportion reporting. The following methods directly parallel the approaches in the main paper with slight modifications to account for the different proportion structure of the forecasting target.

Forecasting

Suppose first that we have an estimate of $p_{ts}(d)$. We will discuss how to estimate this quantity later on. We can implement methods in parallel to those proposed in **Section 3.1** for counts in this setting where the forecast targets are proportions.

- Rescaling: We can use *Eq i* to obtain an estimate of $y_{ts}(\infty)$ in the presence of reporting delay. Then, we can use $y_{ts}(\infty)$ for forecasting.
- Modeling with offset: Suppose our forecast model assumes a logit structure such that $\text{logit}(y_{ts}(\infty)) = f(t, s, X; \theta)$ for some mean model structure $f(t, s, X; \theta)$ possibly a function of covariates X in addition to t and s. In this setting, we can show that

  $$logit\,(y_{ts}(d)) = f(t, s, X; \theta) + \log\left(p_{ts}(d)\right) \qquad (Eq\ l)$$

  In other words, we can fit our forecasting model using $y_{ts}(d)$ instead of $y_{ts}(\infty)$ if we include an offset term, $\log\left(p_{ts}(d)\right)$.
- Imputation: Similar to methods discussed for counts in the main paper, we can obtain multiple imputations of $y_{ts}(\infty)$ using the relationship in *Eq i* and similar logic as in **Section 4**.
- Exclusion: We can exclude the most recent weeks' data as can be done for forecasting case counts

Estimating $p_{ts}(d)$

The above forecasting strategies for accounting for the reporting delay assume that we have an estimate of $p_{ts}(d)$. Here, we describe how we can apply modifications of the methods in **Section 3.2** to estimate $p_{ts}(d)$. First, we note that

$$\begin{aligned}
p_{ts}(d) &= \frac{P(S_{ts}(d) = 1|D_{ts} = 1)}{P(S_{ts}(d) = 1|D_{ts} = 0)} \frac{P(S_{ts}(\infty) = 1|D_{ts} = 0)}{P(S_{ts}(\infty) = 1|D_{ts} = 1)} \qquad (Eq\ m)\\
&= \frac{P(D_{ts} = 1|S_{ts}(d) = 1)P(D_{ts} = 1)}{P(D_{ts} = 0|S_{ts}(d) = 1)P(D_{ts} = 0)} \frac{P(D_{ts} = 0|S_{ts}(\infty) = 1)P(D_{ts} = 0)}{P(D_{ts} = 1|S_{ts}(\infty) = 1)P(D_{ts} = 1)}\\
&= \frac{y_{ts}(d)}{1 - y_{ts}(d)} \frac{1 - y_{ts}(\infty)}{y_{ts}(\infty)}
\end{aligned}$$

We can apply the following approaches to estimate $p_{ts}(d)$:

- Estimated as a function of lag only: Using historical data on real-time case reporting and excluding recent weeks for which $y_{ts}(\infty)$ is not available, we estimate

  $$\hat{p}_{ts}(d) = \text{mean}_{i,j}\left(\frac{y_{ij}(d)}{1 - y_{ij}(d)} \frac{1 - y_{ij}(\infty)}{y_{ij}(\infty)}\right)$$

- Model-based estimation: For each historical week with real-time case reporting (excluding more recent weeks), we can calculate $p_{ij}(d)$ using *Eq m*. Then, we can model $p_{ij}(d)$ as a function of i, j, and possibly additional covariates X. This model can be used to predict the unknown quantity $p_{ts}(d)$ for recent weeks t and s.

- Local estimation: Following logic in the main paper, we can estimate $p_{ts}(d)$ using only the most recent weeks' reporting data as follows:

$$\hat{p}_{ts}(d) = mean_{i=t-K}^{t-d-1} \left(\frac{y_{is}(d)}{1 - y_{is}(d)} \frac{1 - y_{is}(t-i)}{y_{is}(t-i)} \right) \quad (Eq\ n)$$

  Unlike the estimator of $\pi_{ts}(d)$ in *Eq. 8*, this estimator is not necessarily conservative (biased toward 1) for estimating $\hat{p}_{ts}(d)$.

- Sensitivity analysis: We can repeat our analysis using multiple plausible values of $p_{ts}(d)$.

On the potential for bias

We show in the main paper that there can be a substantial bias in forecasting cases when we ignore the reporting delay. Although now shown, our explorations of reporting delay for proportion forecasts show that reporting delay tends to have a much lesser impact on forecast performance for proportions than it does for cases. For forecasting national US ILI, for example, ignoring reporting delay has a comparatively lesser impact on forecasts of $y_{ts}(\infty)$ values compared to its impact on case forecasts $N_{ts}(\infty)$. Still, in settings where $p_{ts}(d)$ is large (>>1) or small (<<1), selection bias caused by lack of representativeness of the intermediate sample of outpatients used to calculate $y_{ts}(d)$ could still have an appreciable impact on forecasts. When historical data on real-time reporting of y is available, we recommend that forecasters evaluate the magnitude of *Eq k* over time when determining whether or not they need correct for reporting delay in forecast modeling.

References

1. Verrall RJ. An investigation into stochastic claims reserving models and the chain-ladder technique. Insurance: Mathematics and Economics. 2000;26(1):91–99. doi:10.1016/S0167-6687(99)00038-4.

2. England PD, Verrall RJ. Stochastic Claims Reserving in General Insurance. British Actuarial Journal. 2002;8(3):443–518. doi:10.1017/s1357321700003809.

3. McGough SF, Johansson MA, Lipsitch M, Menzies NA. Nowcasting by Bayesian smoothing: A flexible, generalizable model for real-time epidemic tracking. PLoS Computational Biology. 2020;16(4):1–20. doi:10.1371/journal.pcbi.1007735.

4. Renshaw AE, Verrall RJ. A stochastic model underlying the chain-ladder technique. British Actuarial Journal. 1998;4(4):903–923.

5. Little RJA, Rubin DB. Statistical analysis with missing data. 2nd ed. Hoboken, NJ: John Wiley and Sons, Inc; 2002.

6. Hohle M, an der Heiden M. Bayesian Nowcasting during the STEC O104: H4 Outbreak in Germany, 2011. Biometrics. 2014;70(4):993–1002. doi:10.1111/biom.l2194.

7. Osthus D. Fast and Accurate Influenza Forecasting in the United States with Inferno. bioRxiv. 2021; p. 1–22.
